# LncRNA OIP5-AS1 knockdown is associated with attenuated nonylphenol-induced cardiac fibrosis

**DOI:** 10.1265/ehpm.25-00436

**Published:** 2026-06-23

**Authors:** Jie He, Jie Xu, Huawen Yu, Ya Luo, Kai Pan, Feng Li, Jie Yu

**Affiliations:** 1 School of Public Health, Zunyi Medical University, Zunyi, Guizhou, 563000, P.R. of China; 2 Guangyuan Center for Disease Control and Prevention, Guangyuan, Sichuan, 628000, P.R. of China

**Keywords:** LncRNA OIP5-AS1, Nonylphenol, H9C2 cells, Cardiac fibrosis, Collagen deposition

## Abstract

**Background:**

Environmental endocrine disruptors (EEDs), endocrine-interfering pollutants, attract attention for potentially affecting heart diseases via epigenetics. Nonylphenol (NP), a classical representative of endocrine-disrupting chemicals, has previously been demonstrated by our research group to induce myocardial fibrosis in rats. LncRNA, a gene regulator, mediates EED-induced heart cell damage and fibrosis.

**Objective:**

This study aimed to investigate the role of long noncoding RNA OIP5 antisense RNA 1 (lncRNA OIP5-AS1) knockdown in NP-induced myocardial fibrosis.

**Methods:**

Small interfering RNA (siRNA) transfection was used to interfere with the expression of lncRNA OIP5-AS1 in H9C2 cells. Lactate dehydrogenase (LDH), cell Counting Kit-8 (CCK-8), transwell, quantitative real time polymerase chain reaction (qRT-PCR), and Western blot assays were employed to detect cell membrane integrity, proliferation, migration ability, and the expression of fibrosis-related genes/proteins in H9C2 cells. This process was also validated in Sprague Dawley (SD) rats.

**Results:**

The expression of fibrosis-related factor mRNA and proteins in H9C2 cells increased, and the expression of lncRNA OIP5-AS1 was significantly upregulated after 24 h of exposure to 70 µmol/L NP. The knockdown of lncRNA OIP5-AS1 did not show differential changes in LDH activity across treatment groups. However, the knockdown of lncRNA OIP5-AS1 significantly attenuated NP-induced proliferation and migration abilities of H9C2 cells and inhibited the increase in the expression of fibrosis-related factor mRNA/proteins caused by NP. This suggested that the interference with lncRNA OIP5-AS1 expression inhibited the NP-induced myocardial fibrosis in H9C2 cells. *In vivo* results suggested that NP exposure and isoproterenol hydrochloride groups in rats showed accumulation of myocardial collagen fibers in the interstitial space, increased distribution and content of collagen fibers, and elevated expression of fibrosis-related proteins. The distribution range, content of collagen fibers, and expression of fibrosis-related proteins significantly reduced in rats with lncRNA OIP5-AS1 knockdown exposed to NP compared with those in the NP group.

**Conclusions:**

The expression of lncRNA OIP5-AS1 was upregulated in NP-treated H9C2 cells, rat hearts, and myocardial fibrosis model rats. Knockdown of lncRNA OIP5-AS1 was associated with attenuated NP-induced myocardial fibrosis and reduced collagen deposition.

**Supplementary information:**

The online version contains supplementary material available at https://doi.org/10.1265/ehpm.25-00436.

## Graphical Abstract

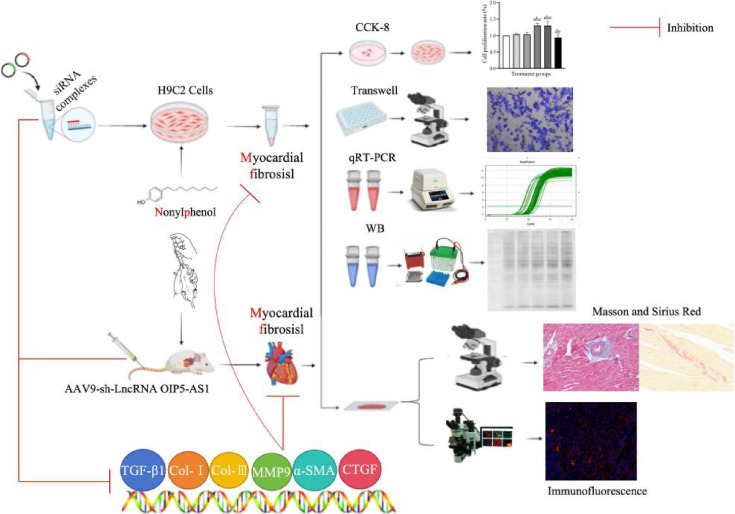

## 1. Introduction

Cardiovascular diseases (CVD) pose a significant threat to global human health. The World Health Organization predicted that CVD mortality would be approximately 26.3 million by 2030 [1]. In China, CVD remains the leading cause of death among urban and rural residents, posing a serious threat to human health [2]. Previous studies detected myocardial fibrosis (MF) in numerous cardiac diseases, including myocardial hypertrophy and myocardial infarction [3–5]. Therefore, investigating the specific mechanisms of MF is crucial for improving the prognosis of heart diseases. The primary pathological feature of MF is the proliferation of fibroblasts in the myocardial stroma, their transformation into myofibroblasts (MyoFB) with enhanced extracellular matrix (ECM) secretion capabilities, and excessive deposition of collagen due to an imbalance in collagen synthesis and degradation [6]. MF is closely related to lifestyle, dietary habits, and environmental factors. Recent studies have focused on the relationship between environmental endocrine disruptors (EEDs) and the pathogenesis of MF. EEDs are exogenous chemicals that disrupt the synthesis, transport, binding, and metabolism of natural hormones, causing irreversible effects on the nervous [7], metabolic [8], reproductive [9], and immune systems [10]. Numerous studies have shown that EEDs can induce cardiac fibrosis. Nonylphenol (NP), a typical EED, has been found to cause cardiac toxicity in rats and activate the transforming growth factor-β1 (TGF-β1)/LIM domain kinase 1 (LIMK1) signaling pathway, promoting MF [11–16].

During MF, transforming growth factor-β1 (TGF-β1) serves as a major regulatory factor that promotes healing in the early stages of cardiac injury. As the primary types of collagen, collagen I and III are induced by TGF-β1 to restore tissue integrity. In the classical TGF-β1/Smads signaling pathway, TGF-β1 activates smad family member 3 (Smad 3) and Smad2 to induce the transformation of cardiac fibroblasts (CFs) into MyoFB, which secrete α-smooth muscle actin (α-SMA) and promote ECM production [17–19]. Additionally, the induction of MF by TGF-β1 involves the complex regulation of interactions between matrix metalloproteinases (MMPs) and tissue inhibitors of metalloproteinases (TIMPs) [20]. Previous studies have found that inhibiting MMP-9 expression can alleviate the severity of MF. Besides TGF-β1, connective tissue growth factor (CTGF) is also considered a pro-fibrotic factor playing a crucial role in the fibrotic process by inducing the growth of MyoFB, accelerating matrix remodeling, and promoting the development of MF [21]. Therefore, TGF-β1, collagen I, collagen III, α-SMA, MMP-9, and CTGF are considered the key markers in fibrosis formation.

Long noncoding RNAs (lncRNAs) are RNAs longer than 200 nucleotides that rarely encode proteins but have regulatory functions. Increasing evidence suggests that lncRNAs regulate the occurrence of fibrosis [22–24]. Additionally, EEDs cause abnormal lncRNA expression, promoting disease progression [25–28]. Our previous sequencing research on CFs exposed to NP revealed the upregulation of lncRNA OIP5-AS1 expression. Literature suggests that lncRNA OIP5-AS1 regulates the progression of various diseases, including heart failure [29], esophageal cancer [30], liver cancer [31], gastric cancer [32], and renal fibrosis [33]. However, its role in MF has not been reported. Thus, we hypothesized that NP exposure upregulated lncRNA OIP5-AS1 expression, promoting TGF-β1 expression, further enhancing collagen and ECM secretion, and ultimately leading to MF. Inhibiting the high expression of lncRNA OIP5-AS1 could reduce collagen and ECM secretion, mitigating NP-induced MF. Therefore, this study combined *in vitro* and *in vivo* experiments to thoroughly investigate the role of lncRNA OIP5-AS1 in NP-induced myocardial fibrosis.

## 2. Methods

### 2.1 Reagents and equipments

The H9C2 cells were purchased from Shanghai Fuheng Biotechnology. Fetal bovine serum was purchased from Sayer Biotechnology (Guangzhou, China; Article No.: FBSST-01033). Nonylphenol (standard, Article No. [84852-15-3]629) was purchased from Dr. Ehrenstorfer GmbH Reagent, Germany. In vitro LncRNA OIP5-AS1 interfering vector siRNA was purchased from Jima Genetics, and in vivo LncRAN OIP5-AS1 interfering vector AAV9 was purchased from Gikai Gene (Shanghai, China). No-StainTm dye-free protein labeling reagent (Item No. A44449) and LipofectamineTM 2000 transfection reagent were purchased from Thermo Fisher Scientific (U.S.A.). Anti-collagen III antibody and Anti-collagen I antibody were purchased from Abcam (Cambridge, UK). Anti-CTGF antibody, Anti-TGF beta 1 antibody, and Anti-α-SMA antibody were purchased from Huaan Bio (Hangzhou, China). PrimeScriptTM RT premix and SYBR^®^ premix Ex TaqTMII were purchased from Takara (Shiga, Japan). All primer sequences were purchased from Shanghai Sangyo. The ECL chemical imaging system, PCR amplifier, and PCR reverse transcriber were purchased from BIO-RAD (California, USA).

### 2.2 *In vitro* experiments

#### 2.2.1 CCK-8 assay to determine the inhibitory effect of NP on the growth of H9C2 cells

Cells (9 × 10^3^/well in 96-well plates) were treated with NP ((0, 20, 40, 60, 80, 100, 120, 140, 160 µM)) at 80–90% confluency. After 24 h, 100 µL of 10% CCK-8 medium was added per well. Following 1.5 h incubation, viability was assessed by measuring A450.

#### 2.2.2 Transfection of lncRNA OIP5-AS1 siRNA into H9C2 cells

Twenty-four hours before transfection, 8 × 10^4^ cells/well were seeded in 12-well plates (targeting 30–50% confluency). For transfection:

(1) Replaced medium with serum/antibiotic-free DMEM (750 µL/well).
(2) Diluted siRNA (100 nM final) in 125 µL Opti-MEM.
(3) Mixed 3 µL Lipofectamine™2000 with 125 µL Opti-MEM (5 min, RT).
(4) Combined siRNA and Lipofectamine mixtures (20 min incubation, RT), mixing gently 3–5 times.
(5) Added complexes to wells (250 µL/well) with gentle mixing.
(6) Cells were incubated (37 °C, 5% CO_2_), refreshed with complete medium after 6 h [34], and harvested at 48 h for qRT-PCR to assess OIP5-AS1 knockdown [35].

#### 2.2.3 Cell grouping and treatment

Cells were divided into six groups: Control, si-NC, si-OIP5-AS1, NP, si-NC+NP, and si-OIP5-AS1+NP.

H9C2 cells were seeded in 6-well plates at 2.3 × 10^5^ cells/well and transfected after 24 hours (si-NC, si-NC+NP, si-OIP5-AS1, and si-OIP5-AS1+NP groups). At 48 hours post-transfection, Control, si-NC and si-OIP5-AS1 groups received fresh complete medium, while NP, si-NC+NP and si-OIP5-AS1+NP groups were treated with 70 µM NP for 24 hours.

#### 2.2.4 LDH release assay to assess cell membrane damage

Cleared supernatants (2000 rpm, 10 min) were assayed per manufacturer’s protocol: wells received ddH_2_O, sodium pyruvate standard (0.2 µmol/mL), samples, substrate buffer, and coenzyme I solution. After mixing and incubation (37 °C, 15 min), reactions with 2,4-dinitrophenylhydrazine (37 °C, 15 min) and 0.4 mol/L NaOH preceded absorbance measurement (450 nm, Thermo Fisher reader) at RT [36].

#### 2.2.5 CCK-8 assay for cell proliferation assessment

Cells seeded in 96-well plates (six groups) underwent respective transfection/NP (60 µM) treatments. After medium removal, 10% CCK-8/medium mixture was added, followed by 1 h incubation (37 °C) and A450 measurement [37].

#### 2.2.6 Transwell assay for cell migration capability

Cells from each group were trypsinized, counted, and adjusted to 1 × 10^5^ cells/mL. The Transwell lower chamber received 700 µL of 20% FBS medium, while 200 µL cell suspension was added to the upper chamber. After overnight incubation, inserts were washed with PBS (3×), gently wiped with cotton swabs, fixed with 4% PFA (15 min), and stained with 10% crystal violet (30 min). Following PBS rinsing, images were acquired using an inverted microscope (OLYMPUS, MODEL BA53 Japan). The protocol was adapted from Tian et al. with modifications [38].

### 2.3 *In vivo* experiments

#### 2.3.1 SD rat grouping and intervention

**Fig. 1:**
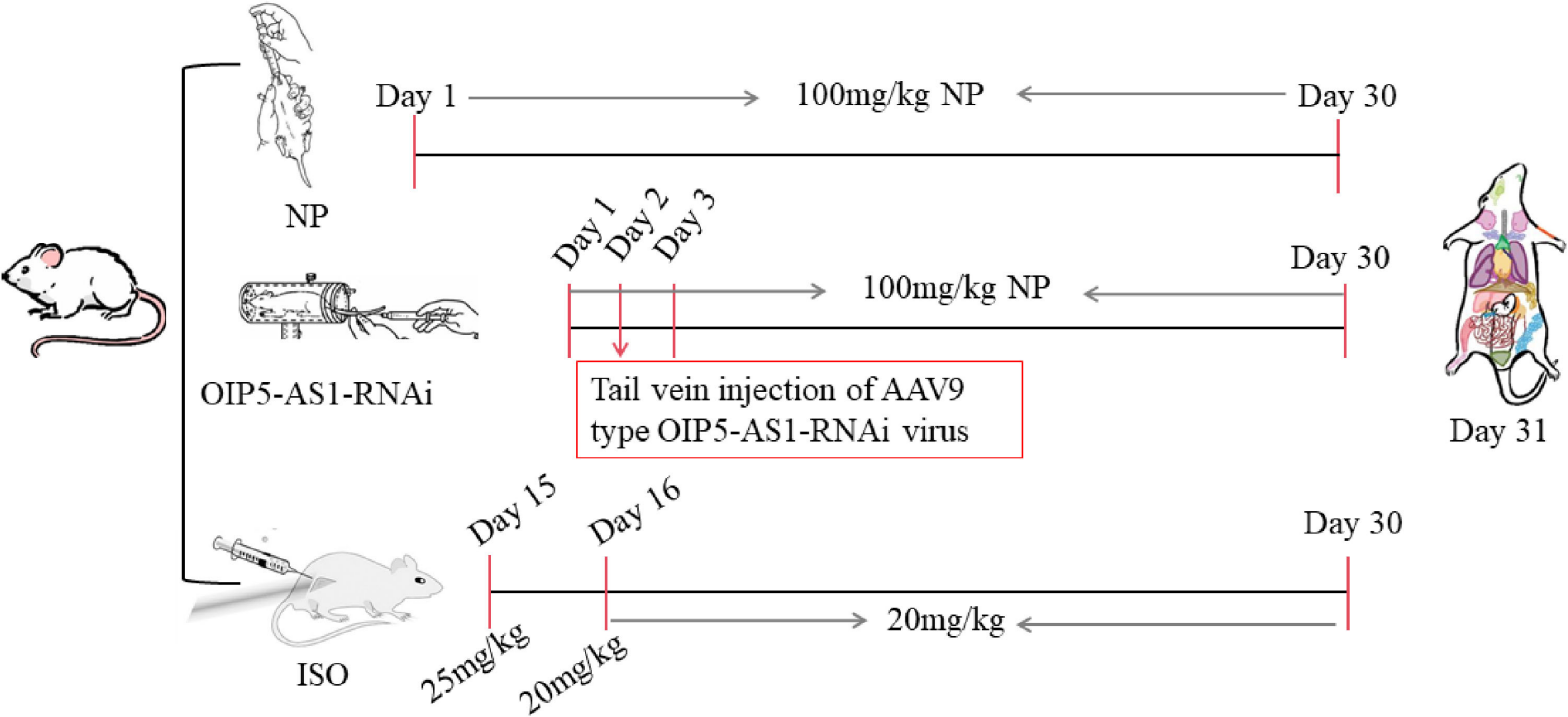
Time stages and processes of rat treatment in each group.

Forty SD rats were randomly divided into five groups (n = 8/group): blank (corn oil), NP (100 mg/kg/d), AAV9-Vector+NP, AAV9-sh-OIP5-AS1+NP, and model (ISO (Isoproterenol hydrochloride)-induced). All groups except model received daily oral NP (100 mg/kg) for 29 days, with AAV9 groups receiving intravenous virus injection (2.3 × 10^12^ vg/mL, 300 µL) on day 2. The model group underwent controlled feeding followed by subcutaneous ISO injections (25 mg/kg initial, 20 mg/kg maintenance, adjusted to 15 mg/kg if needed) from day 15–30 (Fig. 1). After fasting on day 30, tissues were collected on day 31 for qRT-PCR analysis of OIP5-AS1 knockdown.

Previous studies have shown that, after the oral administration of 100 mg/(kg·d) NP for 42 days, collagen fiber arrangement in rat cardiac tissue became loose, partial myocardial fiber structures were damaged, cell nuclei disappeared, and significant inflammatory cell infiltration occurred [39]. Additionally, exposure to 100 mg/(kg·d) NP resulted in MF in rat offspring. Therefore, we chose 100 mg/(kg·d) NP as the dosage for NP exposure in this experiment, with an oral gavage volume of 5 mL/(kg·d), administered continuously for 30 days. The rats were fasted 24 h before sampling, and anesthetized the following day with 20% urethane as an anesthetic agent at a dose of 0.5 mL/100 g via intraperitoneal injection. Animal experiments were approved by the Ethics Committee of Zunyi Medical University [Appl. No: ZMU22-2203-312].

#### 2.3.2 Masson and Sirius red staining to observe collagen deposition in rat cardiac tissue

Rat cardiac tissues were PBS-washed to remove vessels, saline-perfused to eliminate residual blood, and fixed (>24 h). Following graded dehydration (70%→95% ethanol and xylene), tissues were paraffin-embedded and sectioned at 4 µm.

For Masson staining, sections were stained with trichrome solution (10 min), briefly rinsed with distilled water, treated with 1% phosphotungstic acid (5 min) and aniline blue (3 min), differentiated in 1% acetic acid (1 min), then dehydrated through 95%–100% ethanol and xylene. Sirius red staining involved 10 min incubation in saturated picric acid solution, 5 min ethanol rinse (verified microscopically using OLYMPUS, MODEL BA53), 30 min oven-drying at 60 °C, 5 min xylene treatment, and neutral gum mounting. Collagen fibers appeared blue (Masson) or red (Sirius red), with collagen volume fraction quantified using ImageJ by calculating positive staining area versus total area [40].

#### 2.3.3 Immunofluorescence staining of fibrosis-associated proteins (TGF-β1, collagen I, collagen III, α-SMA, and CTGF) in rat cardiac tissue

Following deparaffinization and antigen retrieval, tissue sections were PBS-washed and incubated overnight with primary antibodies (TGF-β1 1:100, collagen I 1:100, collagen III 1:300, α-SMA 1:500, CTGF 1:100). After BSA blocking and PBS washing, sections were treated with secondary antibody, developed with chromogenic substrate, and counterstained with hematoxylin (with blueing rinse). Following graded dehydration, sections were mounted for microscopic analysis [41].

#### 2.3.4 qRT-PCR analysis of the expression of fibrosis-associated genes in the myocardium

Approximately 50 mg of heart tissue was homogenized in 1 mL TRIzol on ice, mixed with 200 µL chloroform, shaken vigorously, and centrifuged (12,000 rpm, 15 min) to collect 300–400 µL supernatant. RNA was precipitated with isopropanol, washed with 75% ethanol, and centrifuged (12,000 rpm, 15 min) before dissolving in 20–50 µL DEPC water. RNA purity was verified (OD260/OD280: 1.9–2.1; OD260/OD230 > 2). The RNA was diluted to 100 ng/µL and reverse-transcribed into cDNA (37 °C for 15 min, 85 °C for 5 s, 4 °C) using a PrimeScriptTM kit. qPCR was performed (95 °C for 30 s pre-denaturation, followed by 40 cycles of 95 °C for 5 s, 60 °C for 30 s, and 72 °C for 15 s) in triplicate, with GAPDH as the reference gene and data analyzed via the 2^−ΔCT^ method [42]. The primer sequences are provided in Table 1.

**Table 1:** Primer sequences.

**Gene name**	**Sequences (5′-3′)**
Collagen I	Forward primer: AGGCGAACAAGGTGACAGAGG
	Reverse primer: GGACCAGCAGGACCACTATCG
Collagen III	Forward primer: AGTCGGAGGAATGGGTGGCTATC
	Reverse primer: CAGGAGATCCAGGATGTCCAGAGG
TGF-β1	Forward primer: GACCGCAACAACGCAATCTATGAC
	Reverse primer: CTGGCACTGCTTCCCGAATGTC
MMP-9	Forward primer: TGTTCGCCTTCTACAGAGGAGACC
	Reverse primer: CTGGTGGGAATGTGTGAGCAAGTC
LncRNA OIP5-AS1	Forward primer: CCCCAGTGAGATGCGGATGTC
	Reverse primer: AGCTGCATCCTCAGGGCTTTTC
GAPDH	Forward primer: GACATGCCGCCTGGAGAAAC
	Reverse primer: AGCCCAGGATGCCCTTTAGT

#### 2.3.5 Western blot (total protein normalization) analysis of the expression of fibrosis-associated proteins (TGF-β1, collagen I, collagen III, α-SMA, and CTGF) in the myocardium

Heart tissue and cells were lysed in cold lysis buffer (high-efficiency protein lysis buffer:PMSF:protease inhibitor = 100:1:1) for 30 min at 4 °C, then centrifuged (12,000 rpm, 30 min) to collect supernatant. Protein concentration was determined by BCA assay, with 20 µg protein loaded for electrophoresis and transferred to PVDF membranes. After No-Stain protein marker visualization, membranes were blocked (rapid blocking solution, 45 min), cut according to target protein molecular weights, and incubated overnight at 4 °C with primary antibodies (collagen I 1:1000, collagen III 1:1000, α-SMA 1:3000, TGF-β1 1:2000, CTGF 1:8000). Following TBST washes, membranes were incubated with secondary antibody (1 h, RT), washed (TBST, 10 min × 3), developed with chemiluminescent substrate, and quantified using Image J [43].

To ensure accurate protein quantification, we employed total protein normalization for cross-group comparisons. Studies suggest that traditional reference proteins (e.g., β-actin, GAPDH) often exhibit variable expression, whereas total protein normalization improves reliability. For instance, automated capillary Western blot analysis of CYP3A4 in 179 liver samples showed stronger protein-mRNA correlation with total protein normalization than with β-actin [44]. Similarly, Gürtler et al. demonstrated that stain-free total protein normalization outperformed GAPDH in sensitivity and robustness for detecting subtle regulatory changes [45]. Thus, we adopted this method to minimize experimental variability and enhance Western blot data accuracy.

#### 2.3.6 Statistical analysis

Statistical analysis was performed using SPSS 29.0 software, and the data were expressed as mean ± standard deviation ( $\bar{X}\pm \text{SD}$ ). The independent-samples t test was used for normally distributed data to compare between two groups. The analysis of variance (ANOVA) was used for comparison between multiple groups. The pairwise comparison between multiple groups was conducted using the Tukey’s Honestly Significant Difference test. For nonhomogeneous variance, logarithmic transformation was used. If the data still did not exhibit homogeneity of variance after transformation, the nonparametric Kruskal–Wallis test was used, Subsequent pairwise comparisons were performed using the Bonferroni method. The significance level was set at α = 0.05. The Western blot band intensity scanning was performed using Image J, and graphs were generated using GraphPad Prism 7.0 and AdobePhotoshop12.0.

## 3. Results

### 3.1 Effects of NP on H9C2 cell viability, and expression levels of fibrosis-related genes (*TGF-β1*, *collagen I*, *collagen III* and *MMP-9*) expression, and LncRNA OIP5-AS1 level

**Fig. 2:**
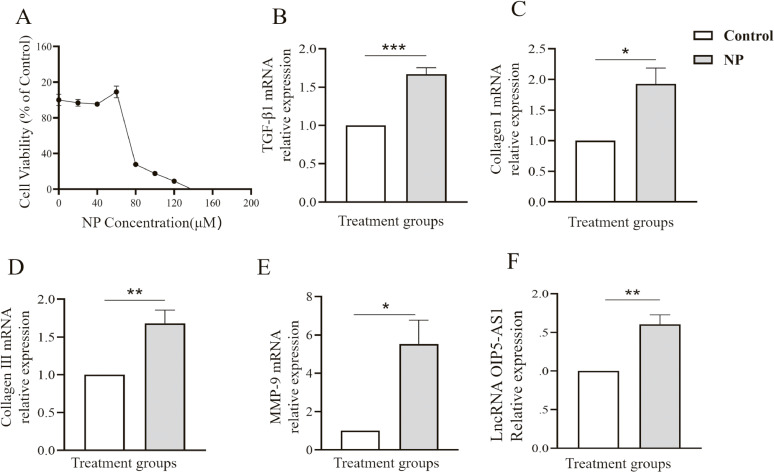
Effects of NP on H9C2 cell viability, and expression levels of fibrosis-related genes and LncRNA OIP5-AS1. A: Impact of NP at different concentration on the viability of H9C2 cells ( $\bar{X}\pm \text{SD}$ , n = 3, Kruskal–Wallis test). B: Relative expression of *TGF-β1* mRNA ( $\bar{X}\pm \text{SD}$ , n = 3, t test) C: Relative expression of *collagen I* mRNA ( $\bar{X}\pm \text{SD}$ , n = 3, t test) D: Relative expression of *collagen III* mRNA ( $\bar{X}\pm \text{SD}$ , n = 3, t test) E: Relative expression of *MMP-9* mRNA ( $\bar{X}\pm \text{SD}$ , n = 3, t test) F: Relative expression of LncRNA OIP5-AS1 ( $\bar{X}\pm \text{SD}$ , n = 3, t test) *P < 0.05; **P < 0.01; ***P < 0.001.

NP exposure dose-dependently reduced H9C2 cell viability over 24 h. At 60 µM, although there was an upward trend in proliferation compared to the control, the difference was not statistically significant (Fig. 2A). With an IC50 of 76.81 µM, 70 µM NP for 24 h was selected for subsequent experiments. An exposure to NP resulted in increased mRNA expression of fibrosis-related genes *TGF-β1* (t = −13.693, *P* < 0.001), *collagen I* (t = −6.176, *P* = 0.025), *collagen III* (t = −26.909, *P* = 0.001) and *MMP-9* (t = −6.363, *P* = 0.024) in H9C2 cells compared with that in the control group (Fig. 2B–2E). Additionally, the expression level of lncRNA OIP5-AS1 in H9C2 cells was upregulated after NP exposure (*P* = 0.001, Fig. 2F).

### 3.2 Effects of NP exposure on the expression of fibrosis-related proteins (TGF-β1, collagen I, collagen III, α-SMA and CTGF) in H9C2 cells

**Fig. 3:**
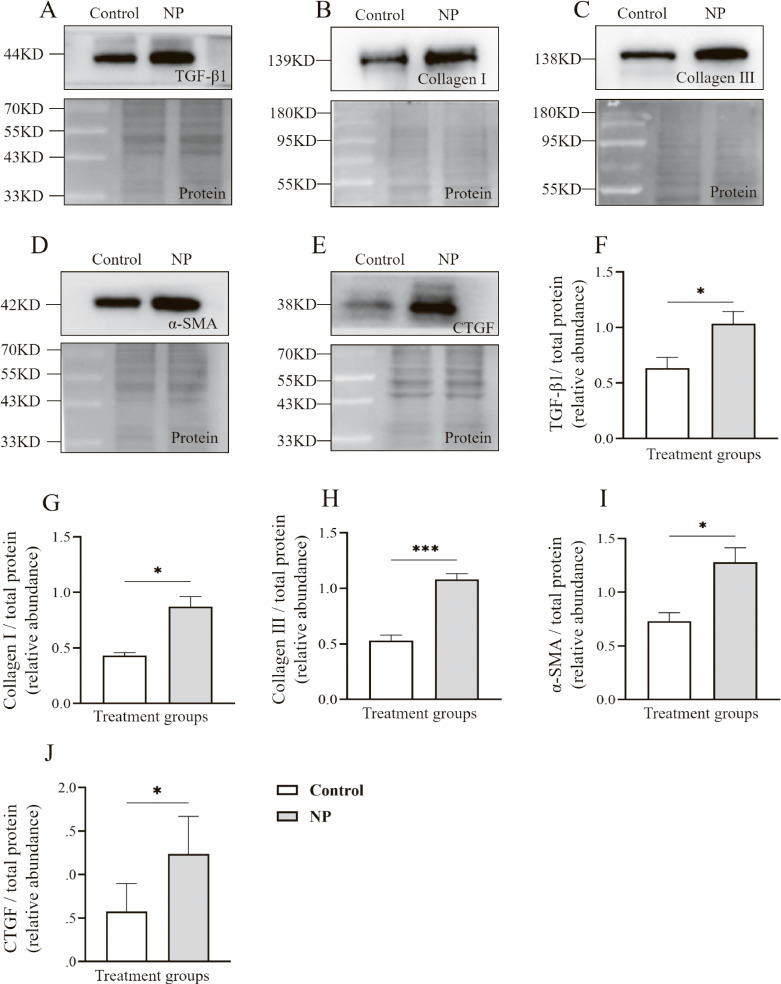
Effects of NP exposure on the expression of fibrosis-related proteins in H9C2 cells. A–E: Fibrosis-related protein bands in Western blot. F: Relative expression of TGF-β1 protein ( $\bar{X}\pm \text{SD}$ , n = 4, t test). G: Relative expression of collagen I protein ( $\bar{X}\pm \text{SD}$ , n = 4, t test). H: Relative expression of collagen III protein ( $\bar{X}\pm \text{SD}$ , n = 4, t test). I: Relative expression of α-SMA protein ( $\bar{X}\pm \text{SD}$ , n = 4, t test). J: Relative expression of CTGF protein ( $\bar{X}\pm \text{SD}$ , n = 4, t test). *P < 0.05; ***P < 0.001.

The protein expression level of TGF-β1 (t = −2.698, *P* = 0.036), collagen I (t = −4.709, *P* = 0.012), collagen III (t = −7.696, *P* < 0.001), α-SMA (t = −3.504, *P* = 0.013) and CTGF (t = −2.459, *P* = 0.049) was elevated in NP-exposed cells compared with that in the control group (Fig. 3A–3J), confirming NP-induced fibrosis in H9C2 cells.

### 3.3 Impact of siRNA on cell membrane integrity and interference effect on lncRNA OIP5-AS1

**Fig. 4:**
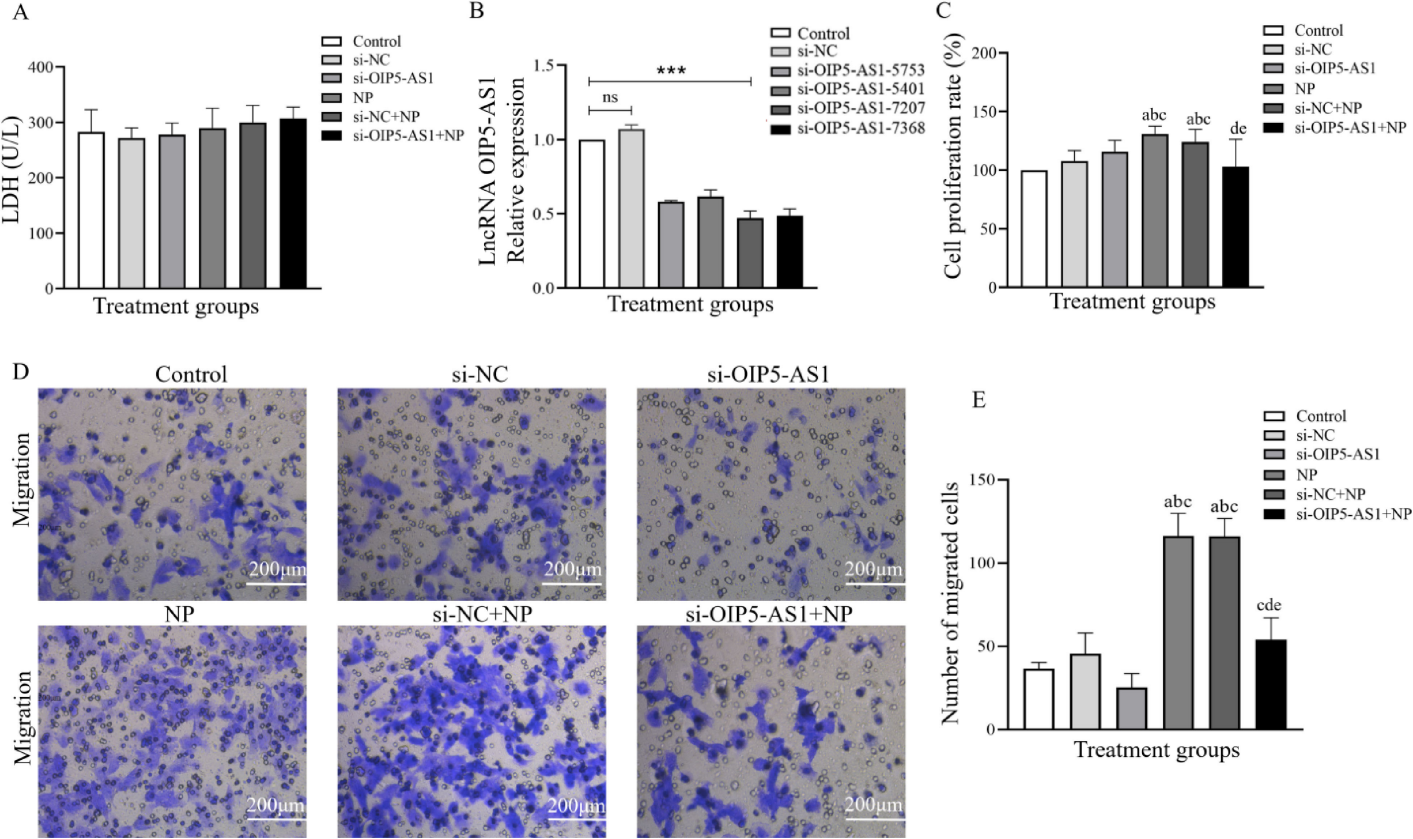
Effects of siRNA and NP on fibrosis in OIP5-AS1-knockdown H9C2 cells. A: Effects of siRNA and NP on cell membrane integrity ( $\bar{X}\pm \text{SD}$ , n = 3, ANOVA). B: siRNA interference effect on lncRNA OIP5-AS1 ( $\bar{X}\pm \text{SD}$ , n = 3, One-way ANOVA followed by Tukey’s HSD test was adopted for statistical analysis). C: Effect of NP on the proliferation of lncRNA OIP5-AS1 knockdown cells ( $\bar{X}\pm \text{SD}$ , n = 3, One-way ANOVA followed by Tukey’s HSD test was adopted for statistical analysis). D: Cell migration in each group. E: Effect of NP on the migration ability of lncRNA OIP5-AS1 knockdown cells ( $\bar{X}\pm \text{SD}$ , n = 4, One-way ANOVA followed by Tukey’s HSD test was adopted for statistical analysis). ns: *P* > 0.05, ***P < 0.001. ^a^vs control group, *P* < 0.05; ^b^vs si-NC group, *P* < 0.05; ^c^vs si-OIP5-AS1 group, *P* < 0.05; ^d^vs NP, *P* < 0.05; ^e^vs si-NC+NP, *P* < 0.05.

No significant changes in LDH activity were observed in the cell culture supernatant after NP and siRNA treatments compared to the control group (*P* = 0.336, Fig. 4-A), indicating that cell membrane integrity remained intact following siRNA transfection and NP exposure, ensuring the reliability of subsequent experiments. The negative control siRNA (si-NC) did not affect lncRNA OIP5-AS1 expression in H9C2 cells. Among the four tested siRNAs, si-OIP5-AS1-Rat-7207 showed the strongest silencing effect (∼53% knockdown, *P* < 0.001, Fig. 4-B) and was therefore selected for further experiments targeting lncRNA OIP5-AS1.

### 3.4 Effects of NP on cell proliferation and migration following knockdown of lncRNA OIP5-AS1

CCK-8 assay revealed that 60 µM NP promoted cell proliferation. Compared to the control, proliferation increased in the NP and si-NC+NP groups but decreased in the si-OIP5-AS1+NP group (*P* = 0.001; Fig. 4C), suggesting OIP5-AS1 mediates NP-induced proliferation. Transwell migration assays showed reduced migration in the si-OIP5-AS1 group versus si-NC. NP enhanced migration in the NP and si-NC+NP groups versus control, whereas si-OIP5-AS1+NP attenuated this effect (*P* < 0.001; Fig. 4D–E). These results indicate that OIP5-AS1 knockdown suppresses NP-induced proliferation and migration in H9C2 cells.

### 3.5 Effects of NP on the expression of fibrosis-related genes and proteins following knockdown of lncRNA OIP5-AS1

**Fig. 5:**
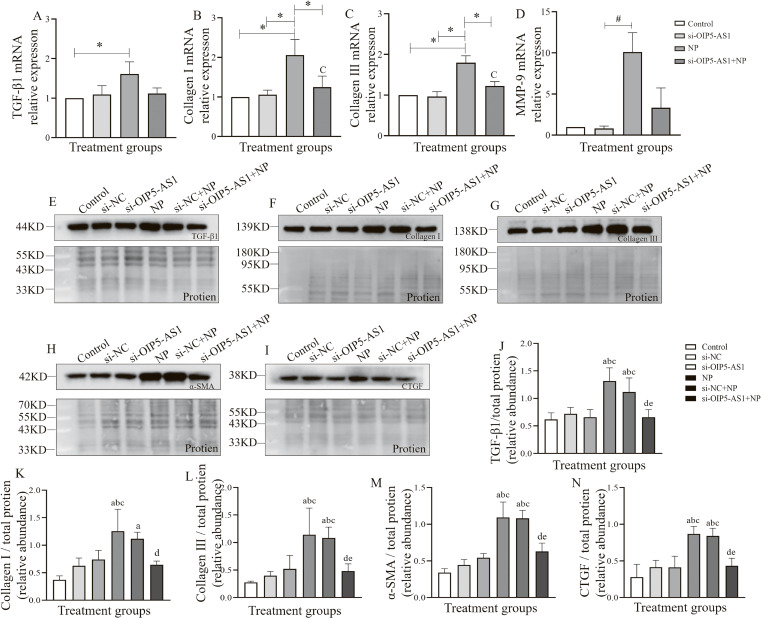
Effects of NP on the expression of fibrosis-related genes and proteins following knockdown of lncRNA OIP5-AS1. A: Relative expression of *TGF-β1* mRNA ( $\bar{X}\pm \text{SD}$ , n = 3, One-way ANOVA followed by Tukey’s HSD test was adopted for statistical analysis). B: Relative expression of *collagen I* mRNA ( $\bar{X}\pm \text{SD}$ , n = 3, One-way ANOVA followed by Tukey’s HSD test was adopted for statistical analysis). C: Relative expression of collagen III mRNA ( $\bar{X}\pm \text{SD}$ , n = 3, One-way ANOVA followed by Tukey’s HSD test was adopted for statistical analysis). D: Relative expression of *MMP-9* mRNA ( $\bar{X}\pm \text{SD}$ , n = 3, Kruskal–Wallis test followed by Bonferroni method was adopted for statistical analysis). E–I: Western blot protein band. J: Relative expression of TGF-β1 protein ( $\bar{X}\pm \text{SD}$ , n = 4, One-way ANOVA followed by Tukey’s HSD test was adopted for statistical analysis). K: Relative expression of collagen I protein ( $\bar{X}\pm \text{SD}$ , n = 4, One-way ANOVA followed by Tukey’s HSD test was adopted for statistical analysis). L: Relative expression of collagen III protein ( $\bar{X}\pm \text{SD}$ , n = 4, One-way ANOVA followed by Tukey’s HSD test was adopted for statistical analysis). M: Relative expression of α-SMA protein ( $\bar{X}\pm \text{SD}$ , n = 4, One-way ANOVA followed by Tukey’s HSD test was adopted for statistical analysis). N: Relative expression of CTGF protein ( $\bar{X}\pm \text{SD}$ , n = 4, One-way ANOVA followed by Tukey’s HSD test was adopted for statistical analysis). * *P* < 0.05; # *P* < 0.05; ^a^vs control group, *P* < 0.05; ^b^vs si-NC group, *P* < 0.05; ^c^vs si-OIP5-AS1 group, *P* < 0.05; ^d^vs NP, *P* < 0.05; ^e^vs si-NC+NP, *P* < 0.05.

Compared with the control group, the NP-treated group exhibited significantly increased mRNA expression of TGF-β1, collagen I, and collagen III. Although MMP-9 expression was also elevated, the increase was not statistically significant. Compared to the NP-treated group, the si-OIP5-AS1 + NP group showed significantly reduced mRNA expression levels of type I collagen and type III collagen. Although TGF-β1 and MMP-9 expression also exhibited decreasing trends, the changes were not statistically significant. (*P*_TGF-β1_ = 0.066; *P*_collagen I_ = 0.022; *P*_collagen III_ = 0.001; *P*_MMP-9_ = 0.05, Fig. 5A–D). The protein analysis revealed that the expression of *TGF-β1*, *collagen I*, *collagen III*, and *CTGF* was elevated in the NP group compared with the control group and decreased in the si-OIP5-AS1+NP group compared with the NP group (*P*_TGF-β1_ = 0.002; *P*_collagen I_ = 0.006; *P*_collagen III_ = 0.015; *P*_α-SMA_ = 0.018; *P*_CTGF_ = 0.004, Fig. 5E–N).

### 3.6 Establishment of myocardial fibrosis model and effect of LncRNA OIP5-AS1 interference

**Fig. 6:**
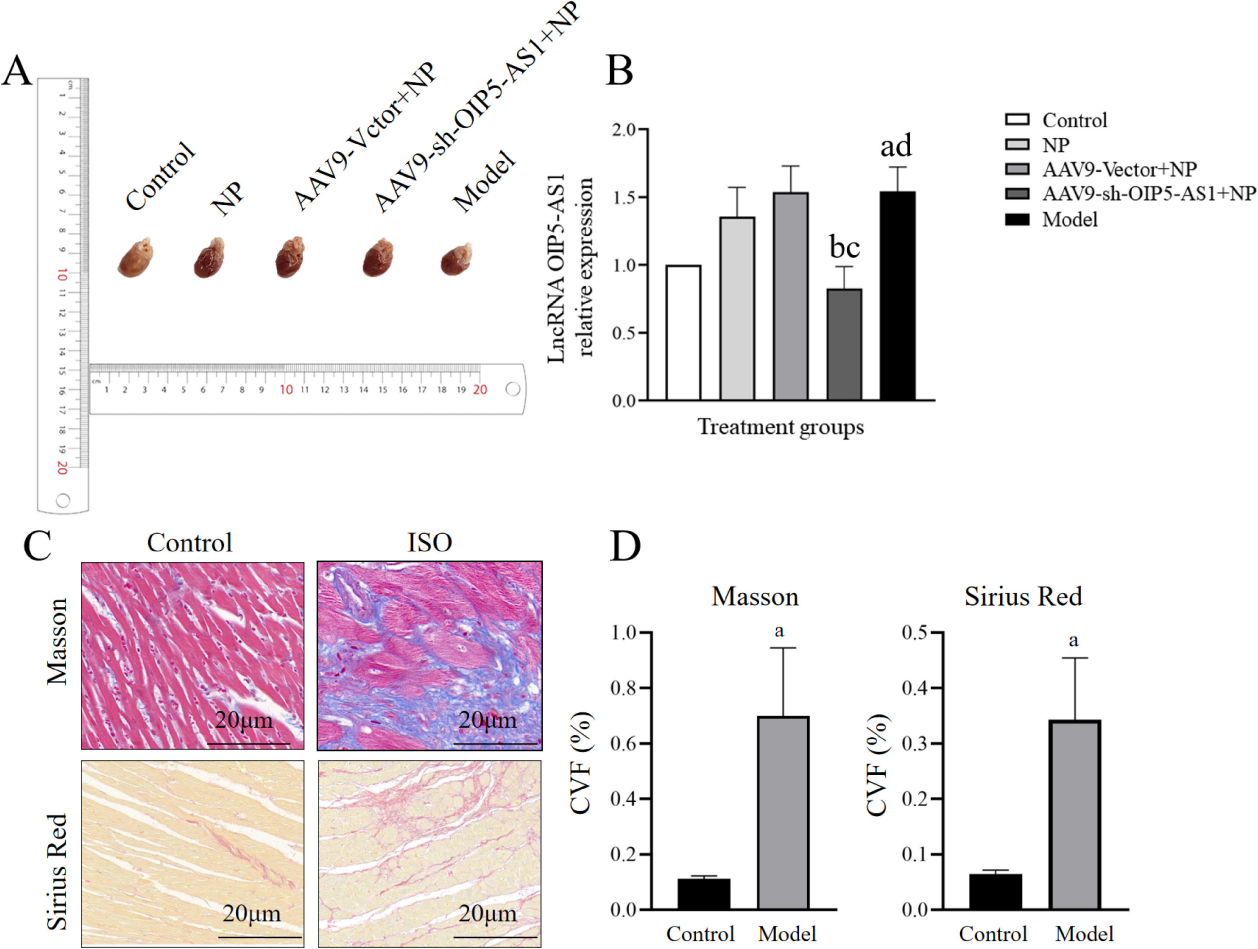
Rat myocardial fibrosis modeling and lncRNA OIP5-AS1 interference detection. A: Naked eye view of rat heart tissue in each group. B: Interference effect of OIP5-AS1-RNAi on the expression of lncRNA OIP5-AS1 in rat heart ( $\bar{X}\pm \text{SD}$ , n = 4, One-way ANOVA followed by Tukey’s HSD test was adopted for statistical analysis). C: Masson and Sirius red staining (scale bar: 20 µm). D: Quantitative analysis of Masson and Sirius red staining ( $\bar{X}\pm \text{SD}$ , n = 3, t test). ^a^vs control group, *P* < 0.05; ^b^vs NP group, *P* < 0.05; ^c^vs AAV9-Vector+NP group, *P* < 0.05; ^d^vs AAV9-sh-OIP5-AS1+NP group, *P* < 0.05.

There was no bleeding point and no obvious abnormality in the heart tissue of each treatment group, and the remaining treatments were darker in color compared with the blank group. (Fig. 6-A). Compared with the blank group, the expression level of LncRNA OIP5-AS1 in the NP group increased by 35%, with no significant statistical difference; however, the LncRNA OIP5-AS1 expression was significantly decreased in the AAV9-sh-OIP5-AS1+NP group relative to the NP group (*P* = 0.021), indicating that rat tail vein injection of AAV9-OIP5-AS1-RNAi was successful in interfering with LncRNA OIP5-AS1 expression (Fig. 6-B). Staining results showed that myocardial fibers in the ISO group were disorganized and filled the interstitial space of cardiac tissues, with a significantly increased distribution range and content (t_Masson_ = −4.154, P = 0.014; t_Sirius Red_ = −4.340, *P* = 0.012) compared with the blank group (Fig. 6-C–D).

### 3.7 Effect of NP on collagen fiber deposition in rat cardiac tissue following knockdown of lncRNA OIP5-AS1

**Fig. 7:**
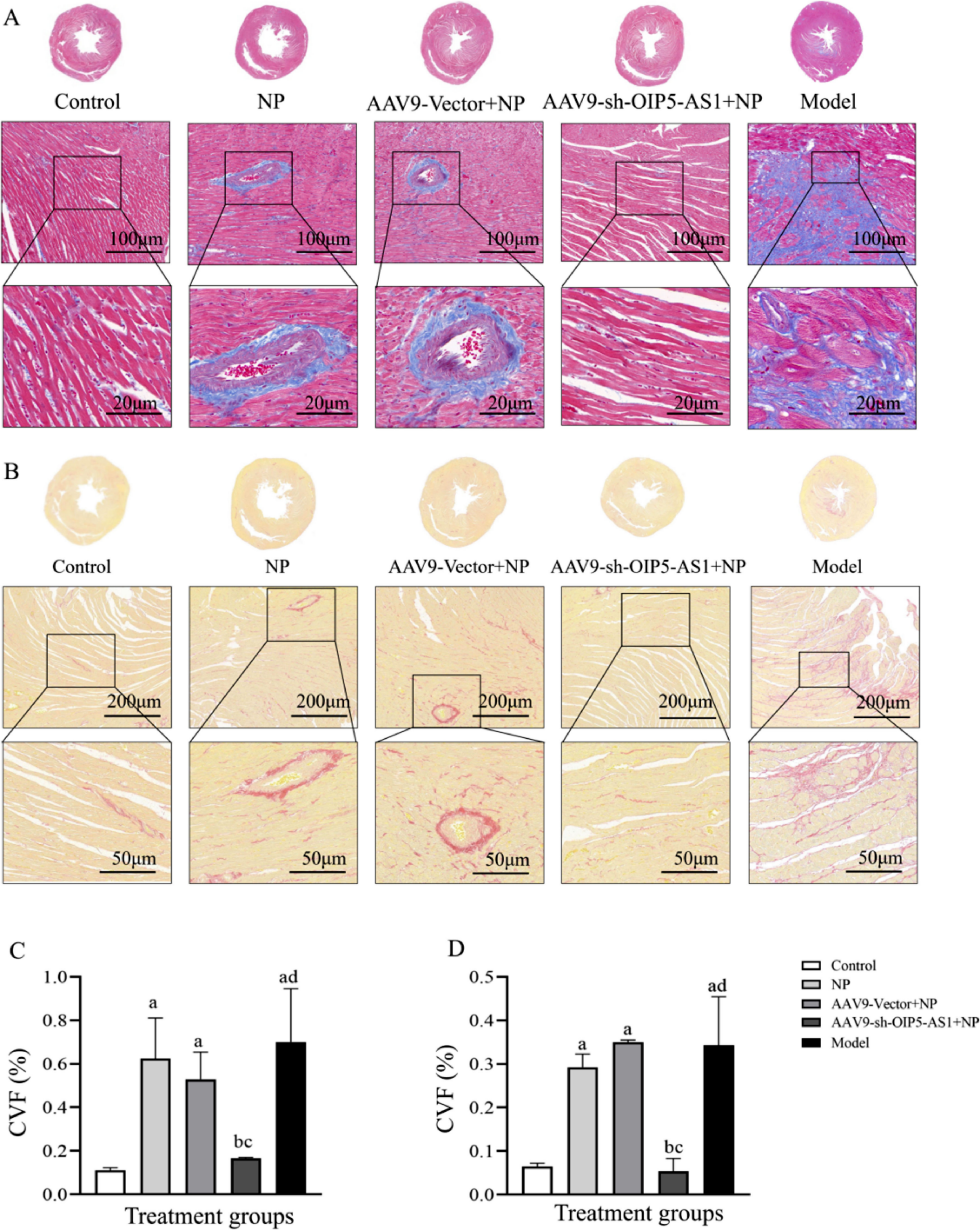
AAV9-mediated OIP5-AS1 knockdown and NP effects on cardiac fibrosis in rats. A: Masson staining of rat heart tissue (collagen fibers in rat heart tissue are blue; muscle fibers, cellulose, and red blood cells are red). B: Sirius red staining of rat heart tissue (collagen fibers in rat heart tissue are red with a yellow background under a light microscope). C: Quantitative analysis of Masson staining (CVF (%) = positive area/total area, $\bar{X}\pm \text{SD}$ , n = 3, One-way ANOVA followed by Tukey’s HSD test was adopted for statistical analysis). D: Quantitative analysis of Sirius red staining (CVF (%) = positive area/total area, $\bar{X}\pm \text{SD}$ , n = 3, One-way ANOVA followed by Tukey’s HSD test was adopted for statistical analysis). ^a^vs control group, *P* < 0.05; ^b^vs NP group, *P* < 0.05; ^c^vs AAV9-Vector+NP group, *P* < 0.05; ^d^vs AAV9-sh-OIP5-AS1+NP group, *P* < 0.05.

Masson and Sirius Red staining revealed extensive blue/red collagen deposition in the NP and AAV9-Vector+NP groups versus control, with disrupted myocardial fiber arrangement. Compared with the NP group, the AAV9-sh-OIP5-AS1+NP group showed reduced collagen fibers and preserved fiber integrity (*P*_Masson_ = 0.002; *P*_Sirius Red_ = 0.001). These results indicated that the *in vivo* interference with lncRNA OIP5-AS1 expression in rats inhibited NP-induced MF (Fig. 7-A–D).

### 3.8 Effect of lncRNA OIP5-AS1 knockdown on NP-induced expression of fibrosis-related proteins in rat myocardium

**Fig. 8:**
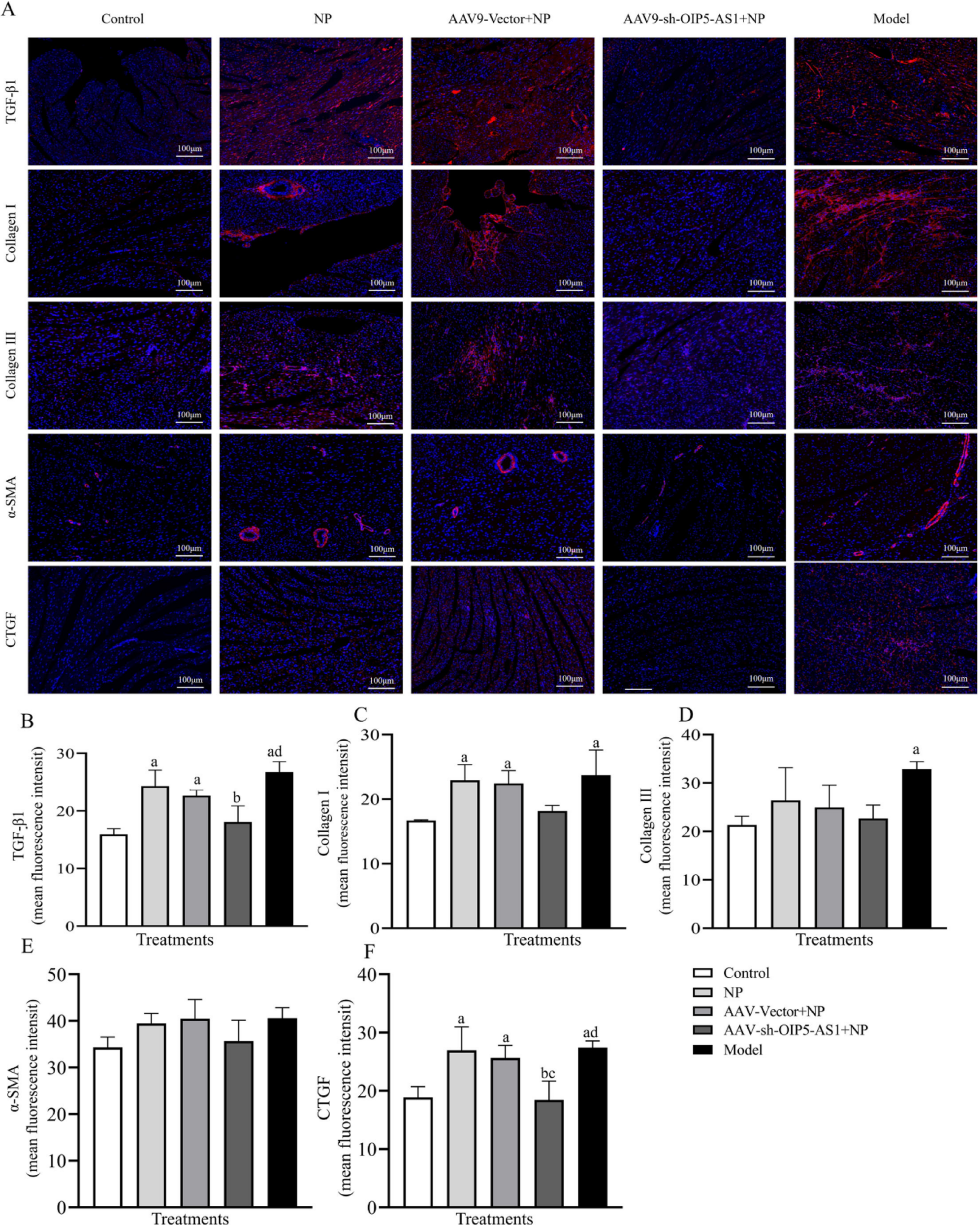
Effect of lncRNA OIP5-AS1 knockdown on NP-induced expression of fibrosis-related proteins in rat myocardium. A: Fluorescence expression of TGF-β1, collagen I, collagen III, α-SMA, and CTGF. B: Fluorescence expression of TGF-β1 ( $\bar{X}\pm \text{SD}$ , n = 3, One-way ANOVA followed by Tukey’s HSD test was adopted for statistical analysis). C: Fluorescence expression of collagen I ( $\bar{X}\pm \text{SD}$ , n = 3, One-way ANOVA followed by Tukey’s HSD test was adopted for statistical analysis). D: Fluorescence expression of collagen III ( $\bar{X}\pm \text{SD}$ , n = 3, One-way ANOVA followed by Tukey’s HSD test was adopted for statistical analysis). E: Fluorescence expression of α-SMA ( $\bar{X}\pm \text{SD}$ , n = 3, One-way ANOVA followed by Tukey’s HSD test was adopted for statistical analysis). F: Fluorescence expression of CTGF ( $\bar{X}\pm \text{SD}$ , n = 3, One-way ANOVA followed by Tukey’s HSD test was adopted for statistical analysis). ^a^vs Control group, *P* < 0.05; ^b^vs NP group, *P* < 0.05; ^c^vs AAV9-Vector+NP group, *P* < 0.05; ^d^vs AAV9-sh-OIP5-AS1+NP group, *P* < 0.05.

Compared with the control group, the expression levels of fibrosis-related markers (TGF-β1, collagen I, CTGF, collagen III, and α-SMA) in cardiac tissues of rats in the NP exposure group and ISO group were generally upregulated, among which the increases in TGF-β1, collagen I and CTGF were statistically significant. After AAV9-sh-OIP5-AS1 intervention, the expression of TGF-β1 and CTGF was markedly inhibited in the AAV9-sh-OIP5-AS1+NP group relative to the NP exposure group. Collagen I, collagen III and α-SMA also showed a downward trend without statistical significance (Fig. 8-A–F).

**Fig. 9:**
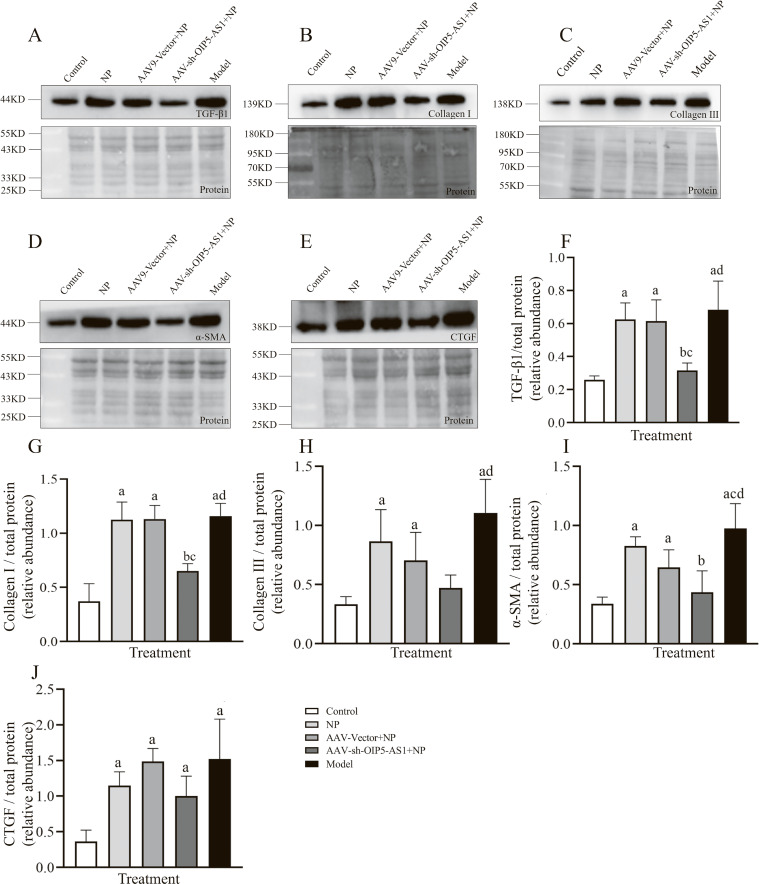
Effect of lncRNA OIP5-AS1 knockdown on NP-induced expression of fibrosis-related proteins in rat myocardium. A–E: Western blot protein band. F: Relative expression of TGF-β1 protein ( $\bar{X}\pm \text{SD}$ , n = 4, One-way ANOVA followed by Tukey’s HSD test was adopted for statistical analysis). G: Relative expression of collagen I ( $\bar{X}\pm \text{SD}$ , n = 4, One-way ANOVA followed by Tukey’s HSD test was adopted for statistical analysis). H: Relative expression of collagen III ( $\bar{X}\pm \text{SD}$ , n = 4, One-way ANOVA followed by Tukey’s HSD test was adopted for statistical analysis). I: Relative expression of α-SMA ( $\bar{X}\pm \text{SD}$ , n = 4, One-way ANOVA followed by Tukey’s HSD test was adopted for statistical analysis). J: Relative expression of CTGF ( $\bar{X}\pm \text{SD}$ , n = 4, One-way ANOVA followed by Tukey’s HSD test was adopted for statistical analysis). ^a^vs Control group, *P* < 0.05; ^b^vs NP group, *P* < 0.05; ^c^vs AAV9-Vector+NP group, *P* < 0.05; ^d^vs AAV9-sh-OIP5-AS1+NP group, *P* < 0.05.

Western blot analysis confirmed that the NP exposure and ISO groups had increased expression of fibrosis-related proteins TGF-β1 (F = 22.942, *P* < 0.001), collagen I (F = 19.745, *P* < 0.001), collagen III (F = 8.448, *P* = 0.001), α-SMA (F = 12.874, *P* < 0.001), and CTGF (F = 9.049, *P* = 0.001) compared with the control group. The AAV9-sh-OIP5-AS1+NP group exhibited lower expression of TGF-β1, collagen I, collagen III, and α-SMA with a decreasing trend in the CTGF protein expression compared with the NP group, but there was no significant difference (Fig. 9-A–J).

## 4. Discussion

### 4.1 Key findings

This study first revealed NP’s effect on lncRNA OIP5-AS1 expression in H9C2 cells using siRNA (in vitro) and AAV9 (in vivo). Our data suggest a potential role for lncRNA OIP5-AS1 in NP-induced myocardial fibrosis. Knockdown of OIP5-AS1 was associated with attenuated NP-triggered proliferation, migration, collagen deposition, and the upregulation of fibrosis-related proteins (TGF-β1, collagen I/III, α-SMA, CTGF). These findings suggest lncRNA OIP5-AS1 as a potential therapeutic target for MF intervention.

### 4.2 Effects of lncRNA OIP5-AS1 knockdown on NP-induced proliferation and migration in H9C2 cells

Previous studies showed that subchronic NP exposure led to MF in rats and their offspring. *In vitro* experiments demonstrated that NP exposure induced fibrosis in primary CFs via the TGF-β1/LIMK1 signaling pathway [15, 16]. Moreover, published studies suggested the involvement of lncRNAs in the pathogenic processes of EEDs [46–49]. However, the involvement of lncRNAs in NP-induced MF was not reported. In this study, we found that NP exposure increased the expression of lncRNA OIP5-AS1 in H9C2 cells and heart tissue of SD rats, suggesting that lncRNA OIP5-AS1 might be a key factor in NP-induced MF. We then used siRNA transfection to knockdown lncRNA OIP5-AS1 expression in H9C2 cells, exposed these cells to NP, and assessed the changes in related indicators to determine whether lncRNA OIP5-AS1 was involved in NP-induced MF. We performed an LDH release assay to verify that NP exposure and transfection did not damage the cell membrane. No significant difference in LDH activity was observed in the culture supernatants of NP-exposed and siRNA-transfected cells compared with the control group, indicating that our exposure and transfection methods did not damage the cell membrane. This ensured the integrity of intracellular substances and improved the reliability of subsequent experimental results.

Ma et al. showed that the *in vitro* knockdown of lncRNA rhabdomyosarcoma 2 associated transcript (RNST) [50], lncRNA PCFL (pro-cardiac fibrotic lncRNA) [51], and lncRNA myosin heavy chain associated RNA transcript (MHRT) [52] significantly inhibited TGF-β1-induced proliferation of CFs. Similarly, we found that the knockdown of lncRNA OIP5-AS1 did not affect the proliferation of cells alone but significantly inhibited the proliferation of NP-exposed lncRNA OIP5-AS1 knockdown cells compared with NP-exposed cells, consistent with previous findings. Furthermore, the knockdown of lncRNAs reduced cell migration, thereby inhibiting disease progression. For instance, lncRNA LITATS1 deficiency promoted TGF-β-induced epithelial–mesenchymal transition (EMT) and migration in cancer cells. The knockdown of LITATS1 effectively enhanced TGF-β/Smads signaling, promoting TGF-β-induced EMT and migration [53]. Similarly, lncRNA-ATB promoted TGF-β-induced glioma cell invasion via the NF-κB and P38/mitogen-activated protein kinase pathways [54]. In our study, the knockdown of lncRNA OIP5-AS1 in H9C2 cells inhibited cell migration compared with that in the si-NC group, consistent with previous findings [55, 56]. The knockdown of lncRNA OIP5-AS1 also inhibited NP-induced cell migration, corroborating findings by Fan et al. [53].

### 4.3 Association of OIP5-AS1 knockdown with attenuated ECM deposition

*In* *vitro* studies indicated that the expression of fibrosis-related genes or proteins did not show any significant changes in the blank control group compared with the control cells. However, the knockdown of lncRNA OIP5-AS1 inhibited the NP-induced increase in the expression of fibrosis-related genes or proteins. These results suggested that lncRNA OIP5-AS1 was a key factor in NP-induced MF, and its knockdown inhibited the fibrosis process in H9C2 cells induced by NP. This conclusion was further validated *in vivo*: the area and number of myocardial interstitial collagen fibers significantly decreased in rats exposed to NP after lncRNA OIP5-AS1 knockdown, and the fluorescence intensity and expression levels of fibrosis-related proteins were reduced. Previous studies reported that interfering with lncRNA expression influenced the fibrosis process [57–61]. Our study demonstrated that the knockdown of lncRNA OIP5-AS1 inhibited NP-induced MF, providing new evidence for this phenomenon. Notably, the involvement of lncRNA OIP5-AS1 in MF was not reported earlier. Although the use of H9C2 cells was considered a shortcoming of this study, the results of the in vitro trial validate the conclusions of the in vivo trial.

### 4.4 Regulatory role of lncRNA OIP5-AS1 in cardiac diseases

Previous studies indicated that high expression of lncRNA OIP5-AS1 promoted the development of heart diseases, and its knockdown alleviated cardiac damage, which was consistent with our findings [62–66]. However, Niu et al. discovered that the overexpression of lncRNA OIP5-AS1 reduced reactive oxygen species–driven mitochondrial damage, thereby decreasing myocardial infarction and apoptosis in H9C2 cells [67]. Similarly, Sun et al. found that the overexpression of lncRNA OIP5-AS1 increased the viability and superoxide dismutase levels of H9C2 cells treated with high glucose, while reducing reactive oxygen species and malondialdehyde levels [68]. Zheng et al. observed that the overexpression of lncRNA OIP5-AS1 inhibited alkaline phosphatase activity and reduced calcified nodule formation, thus inhibiting the development of calcific aortic valve disease [69]. These studies suggested that lncRNA OIP5-AS1 also acted as a “protective” factor, mitigating certain types of cardiac damage. The current research indicated inconsistent roles of lncRNA OIP5-AS1 in different types of heart diseases, highlighting its complex regulatory mechanisms and diverse functions within the body. This suggested that different types of cardiac injuries might activate different signaling pathways. LncRNA OIP5-AS1 might regulate these pathways or factors in various ways, leading to different outcomes.

Furthermore, we found that TGF-β1 was crucial in fibrosis-related diseases, regulating collagen deposition (collagen I) and α-SMA expression during tissue repair. TGF-β1 appeared to be a key factor in regulating fibrosis processes by lncRNA. For instance, lncRNA Gm9866 may regulate liver fibrosis through the TGF-β1 signaling pathway [70]; lncRNA NEAT1 regulates pulmonary fibrosis via the miR-9-5p and TGF-β1 signaling pathways [71]; lncRNA metastasis associated lung adenocarcinoma transcript 1 (MALAT1) is involved in the inhibition of TGF-β1 signaling by melatonin to alleviate MF [72]; and lncRNA PVT1 suppresses TGF-β1 signaling to reduce renal fibrosis [73]. In this study, both *in vitro* and *in vivo* experiments confirmed that NP increased the expression of TGF-β1 and lncRNA OIP5-AS1. Interfering with lncRNA OIP5-AS1 expression reduced the mRNA/protein expression of TGF-β1. Whether the upregulated lncRNA OIP5-AS1 and TGF-β1 jointly promote fibrosis, or whether lncRNA OIP5-AS1 targets and regulates TGF-β1-related signaling pathways to influence fibrosis, needs further investigation.

Although the knockdown efficiency of lncRNA OIP5-AS1 was moderate (∼53%) in our study, a pronounced phenotypic effect was observed. This finding may reflect a nonlinear threshold effect in biological systems. As a regulatory molecule, lncRNA OIP5-AS1 expression may only need to be reduced below a critical threshold to effectively disrupt its downstream profibrotic signaling network (Such as the TGF-β signaling pathway), thereby amplifying the phenotypic outcome. Notably, although functional analyses relied on a single siRNA sequence, the inclusion of negative controls and the consistent observation of molecular changes are consistent with the observed effects being associated with lncRNA OIP5-AS1 knockdown.

### 4.5 Study limitations

While we demonstrated that OIP5-AS1 knockdown (upregulated by NP) attenuates myocardial fibrosis, the effects of OIP5-AS1 overexpression in NP-induced fibrosis remain unknown. Although the observed effects were consistent with the OIP5-AS1 knockdown results, the current data were obtained using only a single siRNA sequence. To further confirm the specificity, additional siRNA sequences or rescue experiments will be applied in subsequent studies to validate these findings. In addition, our data implicate TGF-β1, the exact regulatory mechanism between OIP5-AS1 and TGF-β1 signaling warrants further investigation.

## 5. Conclusions

**Fig. 10:**
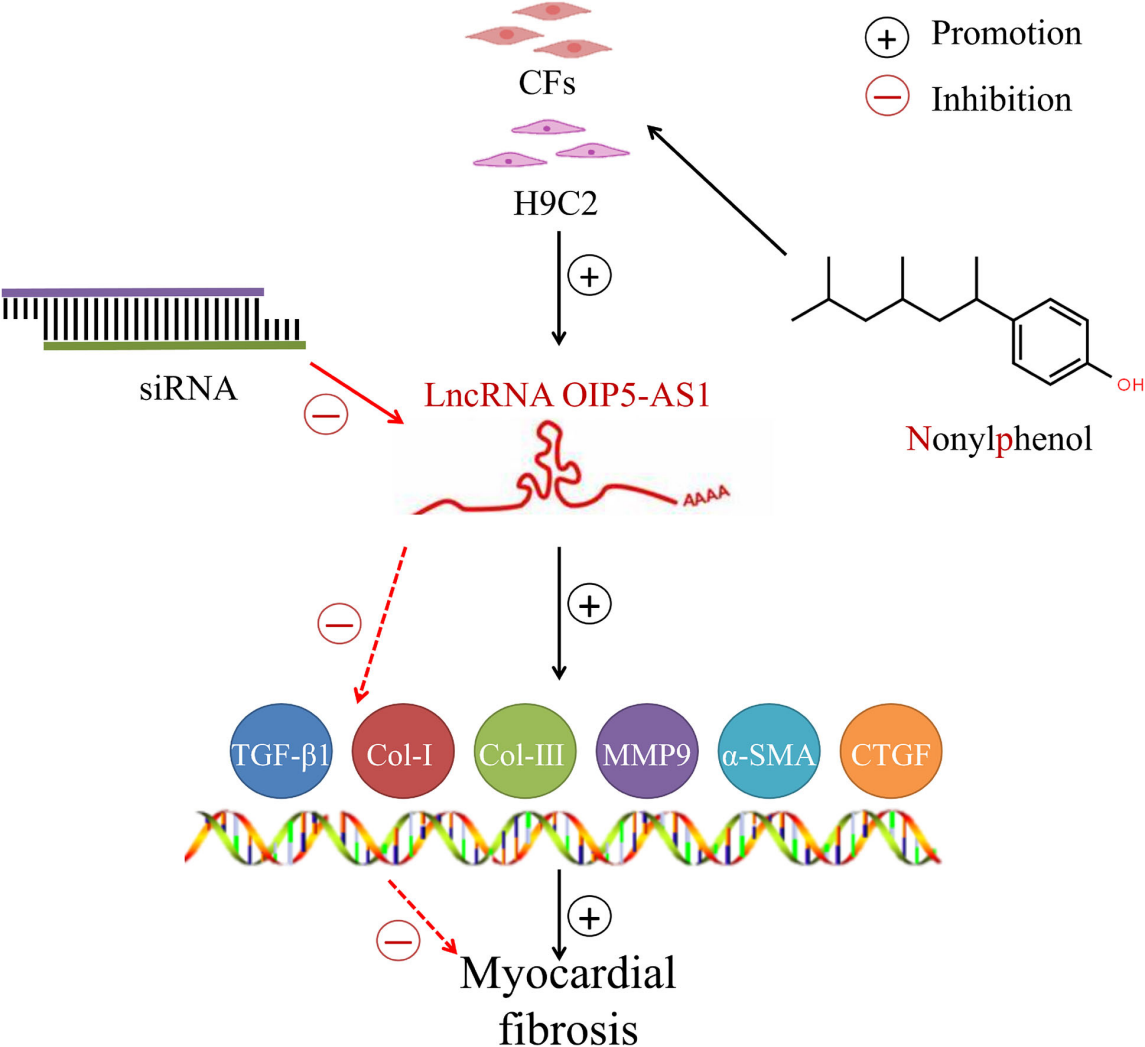
Scientific hypothesis mechanism diagram. The expression of LncRNA OIP5-AS1 was upregulated after NP exposure, which led to an increase in TGF-β1 expression. This further enhanced collagen and ECM secretion, ultimately resulting in the development of myocardial fibrosis. Inhibition of the high expression of LncRNA OIP5-AS1 reduced collagen and ECM secretion and attenuated NP-induced myocardial fibrosis.

LncRNA OIP5-AS1 was upregulated in MF model rats, NP-exposed rats, and H9C2 cells. Knockdown of OIP5-AS1 was associated with attenuated NP-induced proliferation, migration, and collagen deposition in H9C2 cells and suppressed MF in both cellular and rat models (Fig. 10). This study clarifies environmental contributors to MF and explores EED-induced mechanisms, potentially identifying therapeutic targets and biomarkers with clinical significance.

## Declarations

### Availability of data and materials

Relevant data and materials are provided on request.

## References

[1] GBD 2017 Causes of Death Collaborators. Global, regional, and national age-sex-specific mortality for 282 causes of death in 195 countries and territories, 1980–2017: a systematic analysis for the Global Burden of Disease Study 2017. Lancet. 2018;392(10159):1736–88. 30496103 10.1016/S0140-6736(18)32203-7 PMC6227606

[2] Yang J, Zhang Y, Ma T, . Analysis of the Current Status of Cardiovascular Disease Prevalence, Disease Burden and Incidence Forecast in China, 1990–2019. Chinese General Practice. 2024;27(02):233–244+252.

[3] Shivkumar K, Qu Z, Harvey R. Cardiac fibrosis in three dimensions-mechanistic insights into arrhythmic risk due to hypertrophy. J Physiol. 2023;601(2):249–50. 36511350 10.1113/JP283710 PMC9846953

[4] Quagliariello V, De Laurentiis M, Rea D, . The SGLT-2 inhibitor empagliflozin improves myocardial strain, reduces cardiac fibrosis and pro-inflammatory cytokines in non-diabetic mice treated with doxorubicin. Cardiovasc Diabetol. 2021;20(1):150. 34301253 10.1186/s12933-021-01346-y PMC8305868

[5] Yang T, Yang Q, Lai Q, . AP39 inhibits ferroptosis by inhibiting mitochondrial autophagy through the PINK1/parkin pathway to improve myocardial fibrosis with myocardial infarction. Biomed Pharmacother. 2023;165:115195. 37516015 10.1016/j.biopha.2023.115195

[6] Guo Y, Gupte M, Umbarkar P, . Entanglement of GSK-3β, β-catenin and TGF-β1 signaling network to regulate myocardial fibrosis. J Mol Cell Cardiol. 2017;110:109–20. 28756206 10.1016/j.yjmcc.2017.07.011 PMC5581678

[7] Rytel L, Könyves L, Gonkowski S. Endocrine disruptor bisphenol a affects the neurochemical profile of nerve fibers in the aortic arch wall in the domestic pig. Int J Environ Res Public Health. 2022;19(10):5964. 35627499 10.3390/ijerph19105964 PMC9140835

[8] Majewska J, Berg A, Jurewicz J, . Bisphenol A analogues and metabolic syndrome in women with polycystic ovary syndrome. Reprod Toxicol. 2024;123:108511. 37984601 10.1016/j.reprotox.2023.108511

[9] Hwang JM, Bae JW, Lee WJ, . Effect of 4-nonylphenol (4-NP) on sperm function: Insights into the PI3K/PDK1/AKT signaling pathway during capacitation. Reprod Toxicol. 2024;124:108545. 38246476 10.1016/j.reprotox.2024.108545

[10] Popescu M, Feldman TB, Chitnis T. Interplay between endocrine disruptors and immunity: Implications for diseases of autoreactive etiology. Front Pharmacol. 2021;12:626107. 33833678 10.3389/fphar.2021.626107 PMC8021784

[11] Abdelrahman SA, Khattab MA, Youssef MS, . Granulocyte-colony stimulating factor ameliorates di-ethylhexyl phthalate-induced cardiac muscle injury via stem cells recruitment, Desmin protein regulation, antifibrotic and antiapoptotic mechanisms. J Mol Histol. 2023;54(4):349–63. 37428366 10.1007/s10735-023-10137-6 PMC10412672

[12] García-Arévalo M, Lorza-Gil E, Cardoso L, . Ventricular Fibrosis and Coronary Remodeling Following Short-Term Exposure of Healthy and Malnourished Mice to Bisphenol A. Front Physiol. 2021;12:638506. 33912069 10.3389/fphys.2021.638506 PMC8072349

[13] Badawy M, Elsherbiny M, Elshopakey GE, . Potential Effects of Bisphenol A on the Heart and Coronary Artery of Adult Male Rats and the Possible Role of L-Carnitine. J Toxicol. 2022;2022:7760594. 36601412 10.1155/2022/7760594 PMC9807306

[14] Medina-Buelvas DM, Estrada-Muñiz E, Rodríguez-Sosa M, . Increased heart fibrosis and acute infection in a murine Chagas disease model associated with organophosphorus pesticide metabolite exposure. Sci Rep. 2019;9(1):17539. 31772338 10.1038/s41598-019-54218-7 PMC6879754

[15] Liu C, Ni C, Liu W, . Effects of long-term nonylphenol exposure on myocardial fibrosis and cardiac function in rats. Environ Sci Eur. 2021;33:1–13.

[16] Guo M, Xu J, Long X, . Myocardial fibrosis induced by nonylphenol and its regulatory effect on the TGF-β1/LIMK1 signaling pathway. Ecotoxicol Environ Saf. 2024;272:116110. 38364763 10.1016/j.ecoenv.2024.116110

[17] Venugopal H, Hanna A, Humeres C, . Properties and functions of fibroblasts and myofibroblasts in myocardial infarction. Cells. 2022;11(9):1386. 35563692 10.3390/cells11091386 PMC9102016

[18] Lian W, Ge S, Pang Q. Platycodin D ameliorates ammonia-induced pulmonary fibrosis by repressing TGF-β1-mediated extracellular matrix remodeling. Chem Biol Drug Des. 2024;103(1):e14446. 38230787 10.1111/cbdd.14446

[19] Su C, Wang Q, Luo H, . Si-Miao-Yong-An decoction attenuates cardiac fibrosis via suppressing TGF-β1 pathway and interfering with MMP-TIMPs expression. Biomed Pharmacother. 2020;127:110132. 32403042 10.1016/j.biopha.2020.110132

[20] Itzhar A, Yosef G, Eilon-Ashkenazy M, . Potent inhibition of MMP-9 by a novel sustained-release platform attenuates left ventricular remodeling following myocardial infarction. J Control Release. 2023;364:246–60. 37879441 10.1016/j.jconrel.2023.10.033

[21] Song X, Cui Y, Zhu T. MicroRNA-19 upregulation attenuates cardiac fibrosis via targeting connective tissue growth factor. Am J Med Sci. 2023;365(4):375–85. 36539014 10.1016/j.amjms.2022.12.010

[22] Tao H, Cao W, Yang JJ, . Long noncoding RNA H19 controls DUSP5/ERK1/2 axis in cardiac fibroblast proliferation and fibrosis. Cardiovasc Pathol. 2016;25(5):381–9. 27318893 10.1016/j.carpath.2016.05.005

[23] Lang M, Ou D, Liu Z, . LncRNA MHRT promotes cardiac fibrosis via miR-3185 pathway following myocardial infarction. Int Heart J. 2021;62(4):891–9. 34334583 10.1536/ihj.20-298

[24] Ou Y, Liao C, Li H, . LncRNA SOX2OT/Smad3 feedback loop promotes myocardial fibrosis in heart failure. IUBMB Life. 2020;72(11):2469–80. 32959533 10.1002/iub.2375

[25] Yuan L, Tan L, Sun Z, . Plasticizer DEHP exposure in early pregnancy affects the endometrial decidualization in mice through reducing lncRNA RP24-315D19. 10 expression. Zhejiang Da Xue Xue Bao Yi Xue Ban. 2023;52(1):1–12. 37283113 10.3724/zdxbyxb-2022-0669 PMC10407987

[26] Zhang Y, Xie X, Cheng H, . Bisphenol A interferes with lncRNA Fhadlos2 and RUNX3 association in adolescent mouse ovary. Ecotoxicol Environ Saf. 2023;259:115060. 37229876 10.1016/j.ecoenv.2023.115060

[27] Zhang A, Li CY, Kelly EJ, . Transcriptomic profiling of PBDE-exposed HepaRG cells unveils critical lncRNA-PCG pairs involved in intermediary metabolism. PLoS One. 2020;15(2):e0224644. 32101552 10.1371/journal.pone.0224644 PMC7043721

[28] Gao LY, Zhang FQ, Zhao WH, . LncRNA H19 and target gene-mediated cleft palate induced by TCDD. Biomed Environ Sci. 2017;30(9):676–80. 29081343 10.3967/bes2017.090

[29] Zhuang A, Calkin AC, Lau S, . Loss of the long non-coding RNA OIP5-AS1 exacerbates heart failure in a sex-specific manner. iScience. 2021;24(6):102537. 34142046 10.1016/j.isci.2021.102537 PMC8184514

[30] Hou J, Huang Q, Fan Z, . LncRNA OIP5-AS1 knockdown facilitated the ferroptosis and immune evasion by modulating the GPX4 in oesophageal carcinoma. Comput Math Methods Med. 2022;2022:8103198. 35872956 10.1155/2022/8103198 PMC9307385

[31] Shi C, Yang Q, Pan S, . LncRNA OIP5-AS1 promotes cell proliferation and migration and induces angiogenesis via regulating miR-3163/VEGFA in hepatocellular carcinoma. Cancer Biol Ther. 2020;21(7):604–14. 32329664 10.1080/15384047.2020.1738908 PMC7515459

[32] Zhi XH, Jiang K, Ma YY, . OIP5-AS1 promotes the progression of gastric cancer cells via the miR-153-3p/ZBTB2 axis. Eur Rev Med Pharmacol Sci. 2020;24(5):2428–41. 32196594 10.26355/eurrev_202003_20510

[33] Fu JX, Sun GQ, Wang HL, . LncRNA OIP5-AS1 induces epithelial-to-mesenchymal transition and renal fibrosis in diabetic nephropathy via binding to miR-30c-5p. J Biol Regul Homeost Agents. 2020;34(3):961–8. 32519534 10.23812/20-199-A-68

[34] Chen Y, Zhao T, Han M, Chen Y. miR-143 promotes cell proliferation, invasion and migration via directly binding to BRD2 in lens epithelial cells. Am J Transl Res. 2024;16(2):446–57. 38463605 10.62347/BXFG4038 PMC10918123

[35] Zhang K, Zheng X, Sun Y, . TOP2A modulates signaling via the AKT/mTOR pathway to promote ovarian cancer cell proliferation. Cancer Biol Ther. 2024;25(1):2325126. 38445610 10.1080/15384047.2024.2325126 PMC10936659

[36] Kumar P, Nagarajan A, Uchil PD. Analysis of Cell Viability by the Lactate Dehydrogenase Assay. Cold Spring Harb Protoc. 2018;2018(6):10. 10.1101/pdb.prot095497 29858337

[37] Tous C, Muñoz-Redondo C, Gavilán A, . Delving into the Role of lncRNAs in Papillary Thyroid Cancer: Upregulation of LINC00887 Promotes Cell Proliferation, Growth and Invasion. Int J Mol Sci. 2024;25(3):1587. 38338866 10.3390/ijms25031587 PMC10855357

[38] Tian K, Zheng L, Yuan T, . The circRNA hsa-circ-0013561 regulates head and neck squamous cell carcinoma development via the miR-7-5p/PDK3 axis. Cancer Cell Int. 2024;24(1):91. 38429830 10.1186/s12935-024-03256-x PMC10908021

[39] Yang JY, Liu C, Chen NH, . Study of the effects of nonylphenol exposure on myocardial tissue in SD rats. Modern Med Hyg. 2019;35(17):2593–2595+2599.

[40] Li C, Zhang J, Xue M, . SGLT2 inhibition with empagliflozin attenuates myocardial oxidative stress and fibrosis in diabetic mice heart. Cardiovasc Diabetol. 2019;18:1–13. 30710997 10.1186/s12933-019-0816-2 PMC6359811

[41] Wilhelmi T, Xu X, Tan X, . Serelaxin alleviates cardiac fibrosis through inhibiting endothelial-to-mesenchymal transition via RXFP1. Theranostics. 2020;10(9):3905–24. 32226528 10.7150/thno.38640 PMC7086357

[42] Hao K, Lei W, Wu H, . LncRNA-Safe contributes to cardiac fibrosis through Safe-Sfrp2-HuR complex in mouse myocardial infarction. Theranostics. 2019;9(24):7282–97. 31695768 10.7150/thno.33920 PMC6831303

[43] Hagner-McWhirter Å, Laurin Y, Larsson A, Bjerneld EJ, Rönn O. Cy5 total protein normalization in Western blot analysis. Anal Biochem. 2015;486:54–61. 26095394 10.1016/j.ab.2015.06.017

[44] Collins JM, Wang D. Cytochrome P450 3A4 (CYP3A4) protein quantification using capillary western blot technology and total protein normalization. J Pharmacol Toxicol Methods. 2021;112:107117. 34474151 10.1016/j.vascn.2021.107117 PMC8616831

[45] Gürtler A, Kunz N, Gomolka M, . Stain-Free technology as a normalization tool in Western blot analysis. Anal Biochem. 2013;433(2):105–11. 23085117 10.1016/j.ab.2012.10.010

[46] Yuan L, Tan L, Sun Z, . Plasticizer DEHP exposure in early pregnancy affects the endometrial decidualization in mice through reducing lncRNA RP24-315D19. 10 expression. Zhejiang Da Xue Xue Bao Yi Xue Ban. 2023;52(1):1–12. 37283113 10.3724/zdxbyxb-2022-0669 PMC10407987

[47] Zhang Y, Xie X, Cheng H, . Bisphenol A interferes with lncRNA Fhadlos2 and RUNX3 association in adolescent mouse ovary. Ecotoxicol Environ Saf. 2023;259:115060. 37229876 10.1016/j.ecoenv.2023.115060

[48] Zhang A, Li CY, Kelly EJ, . Transcriptomic profiling of PBDE-exposed HepaRG cells unveils critical lncRNA-PCG pairs involved in intermediary metabolism. PLoS One. 2020;15(2):e0224644. 32101552 10.1371/journal.pone.0224644 PMC7043721

[49] Gao LY, Zhang FQ, Zhao WH, . LncRNA H19 and target gene-mediated cleft palate induced by TCDD. Biomed Environ Sci. 2017;30(9):676–80. 29081343 10.3967/bes2017.090

[50] Ma T, Qiu F, Gong Y, . Therapeutic silencing of lncRNA RMST alleviates cardiac fibrosis and improves heart function after myocardial infarction in mice and swine. Theranostics. 2023;13(11):3826–43. 37441584 10.7150/thno.82543 PMC10334841

[51] Sun F, Zhuang Y, Zhu H, . LncRNA PCFL promotes cardiac fibrosis via miR-378/GRB2 pathway following myocardial infarction. J Mol Cell Cardiol. 2019;133:188–98. 31220469 10.1016/j.yjmcc.2019.06.011

[52] Lang M, Ou D, Liu Z, . LncRNA MHRT Promotes Cardiac Fibrosis via miR-3185 Pathway Following Myocardial Infarction. Int Heart J. 2021;62(4):891–9. 34334583 10.1536/ihj.20-298

[53] Fan C, Wang Q, Kuipers TB, . LncRNA LITATS1 suppresses TGF-β-induced EMT and cancer cell plasticity by potentiating TβRI degradation. EMBO J. 2023:e112806. 36994542 10.15252/embj.2022112806 PMC10183827

[54] Tang F, Wang H, Chen E, . LncRNA-ATB promotes TGF-β-induced glioma cells invasion through NF-κB and P38/MAPK pathway. J Cell Physiol. 2019;234(12):23302–14. 31140621 10.1002/jcp.28898

[55] Xie RJ. m6A-modified lncRNA OIP5-AS1 promotes proliferation, migration, invasion, and glycolysis in gastric cancer[D]. Medical University of the South. 2023.

[56] Zeng HJ. Role of LncRNA OIP5-AS1/microRNA-129-5p/SOX2 axis in malignant phenotype and adriamycin resistance of breast cancer and its mechanisms[D]. Nanjing University. 2018.

[57] Yang L, Zheng S, Ge D, . LncRNA-COX2 inhibits Fibroblast Activation and Epidural Fibrosis by Targeting EGR1. Int J Biol Sci. 2022;18(4):1347–62. 35280679 10.7150/ijbs.67974 PMC8898373

[58] Yang L, Deng J, Ma W, . Ablation of lncRNA Miat attenuates pathological hypertrophy and heart failure. Theranostics. 2021;11(16):7995–8007. 34335976 10.7150/thno.50990 PMC8315059

[59] Wu M, Sun J, Wang L, . The lncRNA HOTAIR via miR-17-5p is involved in arsenite-induced hepatic fibrosis through regulation of Th17 cell differentiation. J Hazard Mater. 2023;443:130276. 36332283 10.1016/j.jhazmat.2022.130276

[60] Han X, Guo B, Zhao S, . lncRNA Helf promotes hepatic inflammation and fibrosis by interacting with PTBP1 to facilitate PIK3R5 mRNA stabilization. Cell Mol Biol Lett. 2023;28(1):77. 37805473 10.1186/s11658-023-00492-3 PMC10560431

[61] Peng T, Liu M, Hu L, . LncRNA Airn alleviates diabetic cardiac fibrosis by inhibiting activation of cardiac fibroblasts via a m6A-IMP2-p53 axis. Biol Direct. 2022;17(1):1–22. 36384975 10.1186/s13062-022-00346-6 PMC9670606

[62] Ma J, Qian H, Zou H. Suppression of lncRNA OIP5-AS1 Attenuates Apoptosis and Inflammation, and Promotes Proliferation by Mediating miR-25-3p Expression in Lipopolysaccharide-Induced Myocardial Injury. Anal Cell Pathol. 2023;2023:3154223. 10.1155/2023/3154223 PMC10042636 36994450

[63] Yue Q, Liu Y, Ji J, . Down-regulation of OIP5-AS1 inhibits obesity-induced myocardial pyroptosis and miR-22/NLRP3 inflammasome axis. Immun Inflamm Dis. 2023;11(10):e1066. 37904706 10.1002/iid3.1066 PMC10611552

[64] Dong H, Jiang G, Zhang J, . LncRNA OIP5-AS1 promotes the proliferation and migration of vascular smooth muscle cells via regulating miR-141-3p/HMGB1 pathway. Am J Med Sci. 2022;363(6):538–47. 35278365 10.1016/j.amjms.2022.02.012

[65] Ren M, Wang T, Han Z, . Long noncoding RNA OIP5-AS1 contributes to the progression of atherosclerosis by targeting miR-26a-5p through the AKT/NF-κB pathway. J Cardiovasc Pharmacol. 2020;76(5):635–44. 32833899 10.1097/FJC.0000000000000889

[66] Wang M, Liu Y, Li C, . Long noncoding RNA OIP5-AS1 accelerates the ox-LDL mediated vascular endothelial cells apoptosis through targeting GSK-3β via recruiting EZH2. Am J Transl Res. 2019;11(3):1827. 30972206 PMC6456540

[67] Niu X, Pu S, Ling C, . lncRNA Oip5-as1 attenuates myocardial ischaemia/reperfusion injury by sponging miR-29a to activate the SIRT1/AMPK/PGC1α pathway. Cell Prolif. 2020;53(6):e12818. 32468629 10.1111/cpr.12818 PMC7309946

[68] Sun H, Wang C, Zhou Y, . Long noncoding RNA OIP5-AS1 overexpression promotes viability and inhibits high glucose-induced oxidative stress of cardiomyocytes by targeting microRNA-34a/SIRT1 axis in diabetic cardiomyopathy. Endocr Metab Immune Disord Drug Targets. 2021;21(11):2017–27. 33380310 10.2174/1871530321666201230090742

[69] Zheng D, Wang B, Zhu X, . LncRNA OIP5-AS1 inhibits osteoblast differentiation of valve interstitial cells via miR-137/TWIST11 axis. Biochem Biophys Res Commun. 2019;511(4):826–32. 30846207 10.1016/j.bbrc.2019.02.109

[70] Liao X, Ruan X, Yao P, . LncRNA-Gm9866 promotes liver fibrosis by activating TGFβ/Smad signaling via targeting Fam98b. J Transl Med. 2023;21(1):778. 37919785 10.1186/s12967-023-04642-1 PMC10621198

[71] Zhang Y, Yao XH, Wu Y, . LncRNA NEAT1 regulates pulmonary fibrosis through miR-9-5p and TGF-β signaling pathway. Eur Rev Med Pharmacol Sci. 2020;24(16):8483–92. 32894569 10.26355/eurrev_202008_22661

[72] Che H, Wang Y, Li H, . Melatonin alleviates cardiac fibrosis via inhibiting lncRNA MALAT1/miR-141-mediated NLRP3 inflammasome and TGF-β1/Smads signaling in diabetic cardiomyopathy. FASEB J. 2020;34(4):5282–98. 32067273 10.1096/fj.201902692R

[73] Cao L, Qin P, Zhang J, . LncRNA PVT1 suppresses the progression of renal fibrosis via inactivation of TGF-β signaling pathway. Drug Des Devel Ther. 2020:3547–57. 10.2147/DDDT.S245244 PMC7457787 32921988

