# Supplementary material for: LncRNA OIP5-AS1 knockdown is associated with attenuated nonylphenol-induced cardiac fibrosis
Source: Environ Health Prev Med. 2026 Jun 23;31:39. doi: 10.1265/ehpm.25-00436 (PMC13366185; doi:10.1265/ehpm.25-00436)

The following images are in order: uncut Western blots, blots in the manuscript, and complete gels.

1. Comparison of protein expression between Control and NP groups

TGF-β1 ：

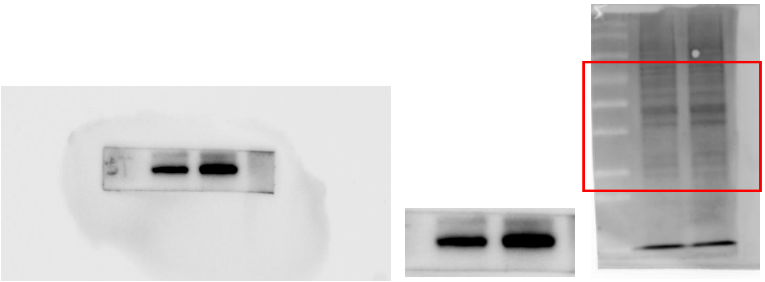

Collagen Ⅰ：

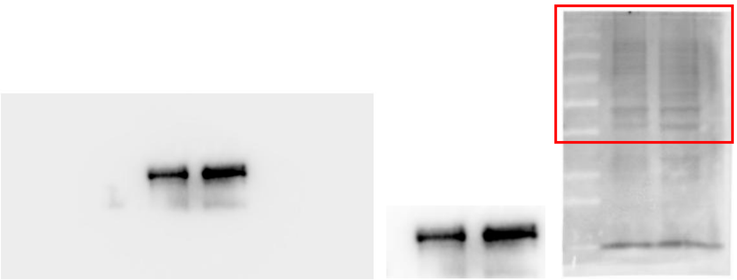

Collagen III：

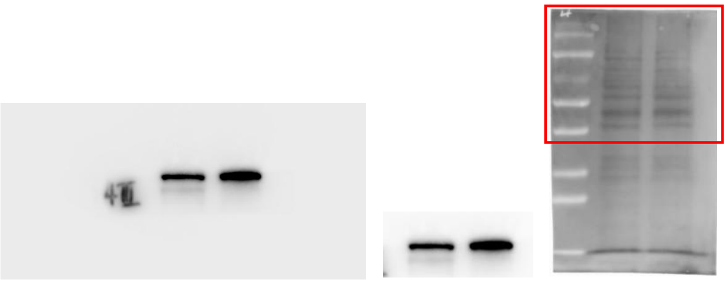

α-SMA：

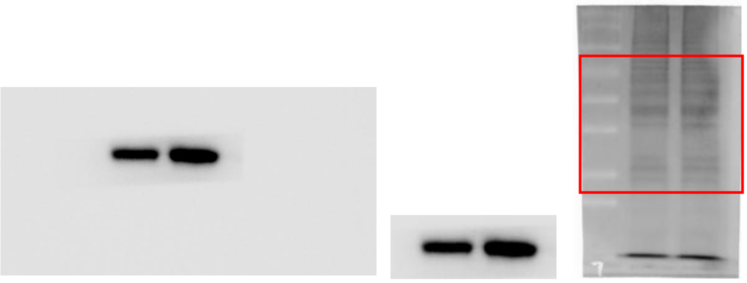

CTGF：

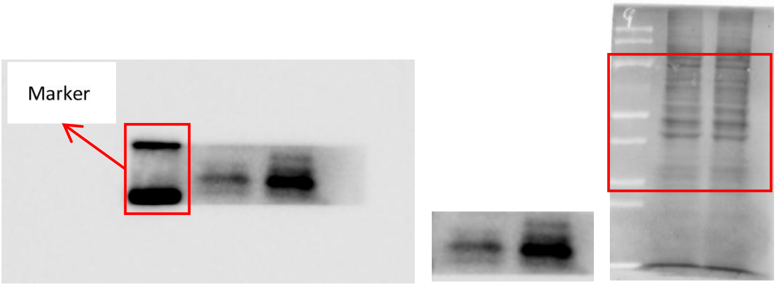

2. Comparison of protein expression levels of Control, si-NC, si-OIP5-AS1, NP, si-NC+NP, si-OIP5-AS1+NP groups

TGF-β1 ：

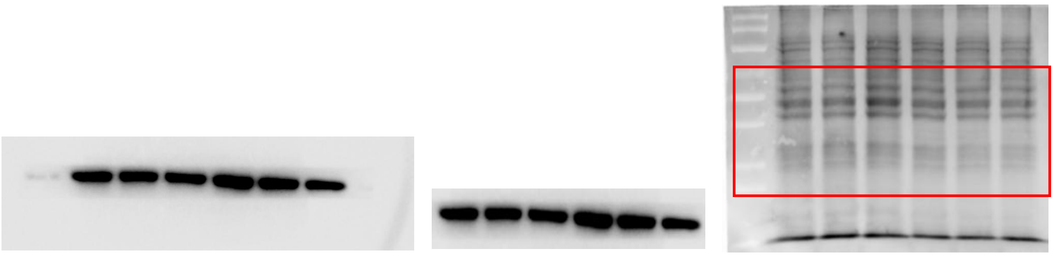

Collagen Ⅰ ：

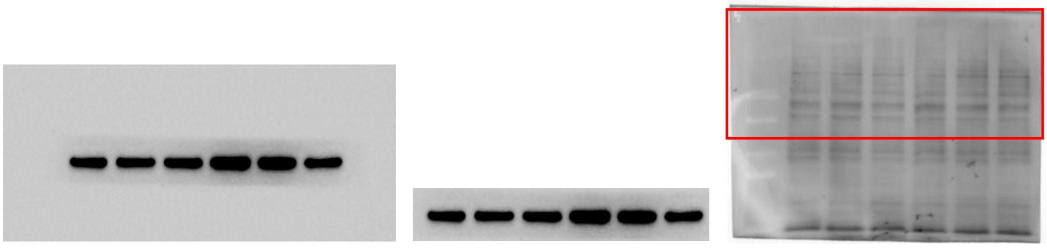

Collagen Ⅲ ：

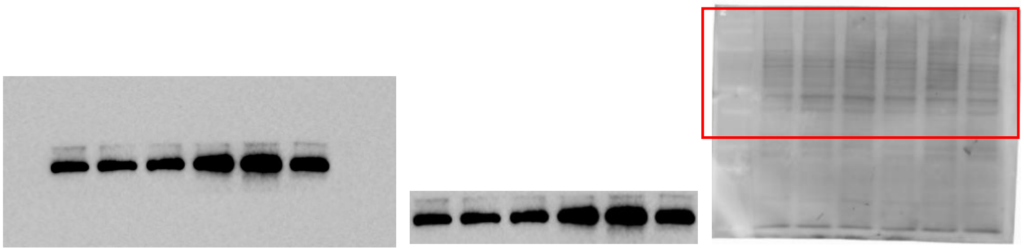

α-SMA ：

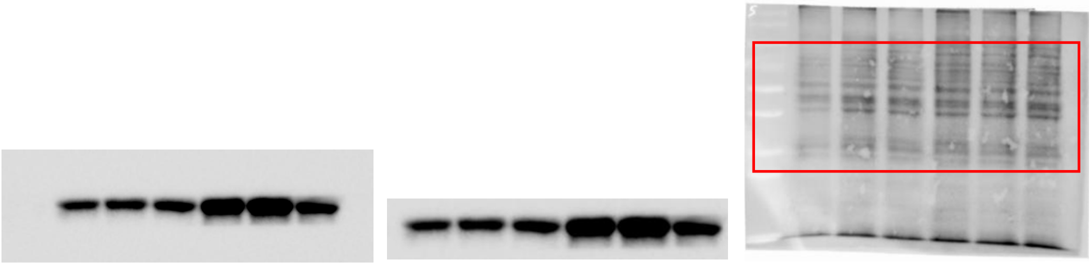

CTGF ：

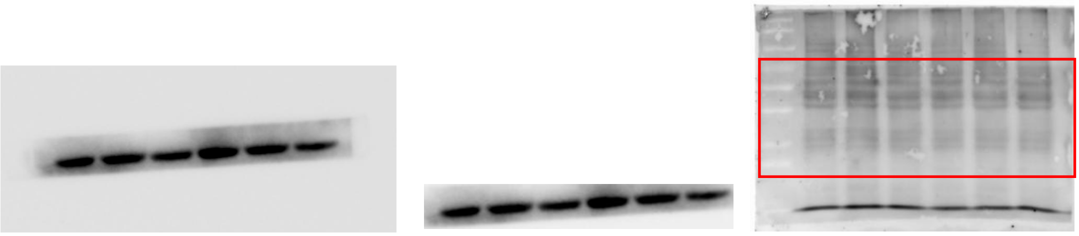

3. Comparison of histone expression levels between Control, NP, AAV9-Vector+NP, AAV9-sh-OIP5-AS1+NP, and Model groups

TGF-β1 ：

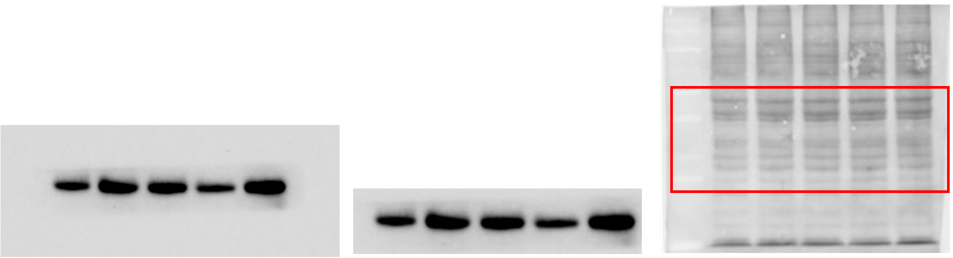

Collagen Ⅰ：

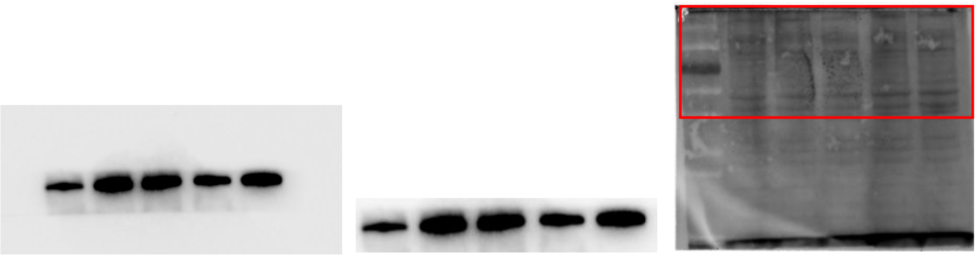

Collagen III：

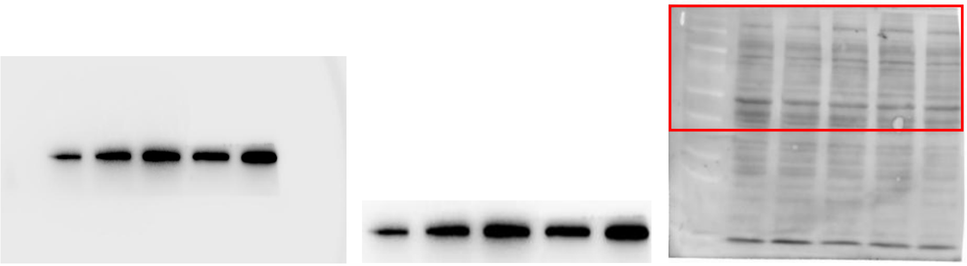

α-SMA

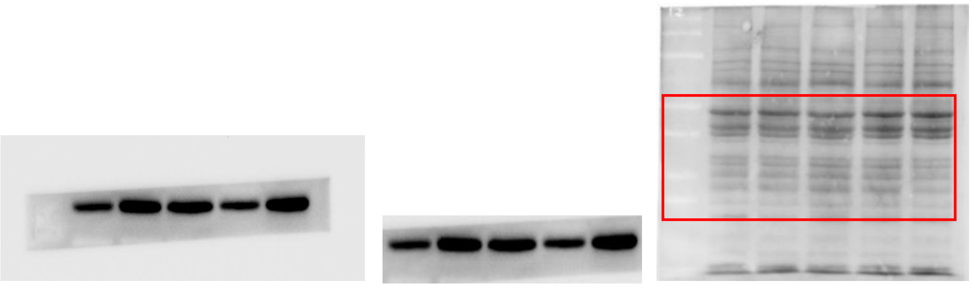

CTGF

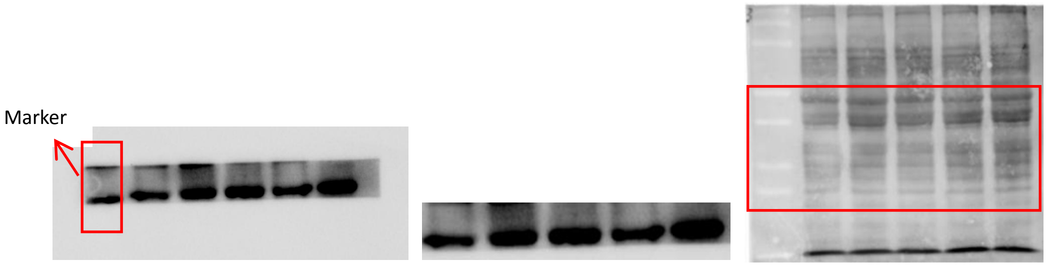

Supplement: Supplementary file 1 — Additional file 1: Supplementary material. [file ehpm-31-039-s001.pdf]
